# Evolving outcomes of extracorporeal membrane oxygenation during the first 2 years of the COVID-19 pandemic: a systematic review and meta-analysis

**DOI:** 10.1186/s13054-022-04011-2

**Published:** 2022-05-23

**Authors:** Ryan Ruiyang Ling, Kollengode Ramanathan, Jackie Jia Lin Sim, Suei Nee Wong, Ying Chen, Faizan Amin, Shannon M. Fernando, Bram Rochwerg, Eddy Fan, Ryan P. Barbaro, Graeme MacLaren, Kiran Shekar, Daniel Brodie

**Affiliations:** 1 Yong Loo Lin School of Medicine, National University of Singapore, National University Health System, Singapore, Singapore; 2 Cardiothoracic Intensive Care Unit, National University Heart Centre, National University Hospital, National University Health System, Level 9, 1E Kent Ridge Road, Singapore, Singapore 119228; 3 Medical Resource Team, National University of Singapore Libraries, Singapore, Singapore; 4 Agency for Science, Technology and Research, Singapore, Singapore; 5 Department of Medicine, Division of Critical Care, McMaster University, Hamilton, ON Canada; 6 Division of Critical Care, Department of Medicine, University of Ottawa, Ottawa, ON Canada; 7 Department of Health Research Methods, Evidence and Impact, McMaster University, Hamilton, ON Canada; 8 Interdepartmental Division of Critical Care Medicine, University of Toronto, Toronto, Canada; 9 Division of Paediatrics Critical Care Medicine, University of Michigan, Ann Arbor, MI USA; 10 Child Health Evaluation and Research Center, University of Michigan, Ann Arbor, MI USA; 11 Adult Intensive Care Services, Prince Charles Hospital, Brisbane, QLD Australia; 12 Queensland University of Technology, Brisbane, Australia; 13 University of Queensland, Brisbane and Bond University, Gold Coast, QLD Australia; 14 Department of Medicine, Columbia University College of Physicians and Surgeons, New York, NY USA; 15 Center for Acute Respiratory Failure, New York-Presbyterian Hospital, New York, NY USA

**Keywords:** Extracorporeal membrane oxygenation, Coronavirus disease 2019, Severe acute respiratory syndrome coronavirus 2, Mortality, Meta-analysis

## Abstract

**Background:**

Extracorporeal membrane oxygenation (ECMO) has been used extensively for coronavirus disease 2019 (COVID-19)-related acute respiratory distress syndrome (ARDS). Reports early in the pandemic suggested that mortality in patients with COVID-19 receiving ECMO was comparable to non-COVID-19-related ARDS. However, subsequent reports suggested that mortality appeared to be increasing over time. Therefore, we conducted an updated systematic review and meta-analysis, to characterise changes in mortality over time and elucidate risk factors for poor outcomes.

**Methods:**

We conducted a meta-analysis (CRD42021271202), searching MEDLINE, Embase, Cochrane, and Scopus databases, from 1 December 2019 to 26 January 2022, for studies reporting on mortality among adults with COVID-19 receiving ECMO. We also captured hospital and intensive care unit lengths of stay, duration of mechanical ventilation and ECMO, as well as complications of ECMO. We conducted random-effects meta-analyses, assessed risk of bias of included studies using the Joanna Briggs Institute checklist and evaluated certainty of pooled estimates using GRADE methodology.

**Results:**

Of 4522 citations, we included 52 studies comprising 18,211 patients in the meta-analysis. The pooled mortality rate among patients with COVID-19 requiring ECMO was 48.8% (95% confidence interval 44.8–52.9%, high certainty). Mortality was higher among studies which enrolled patients later in the pandemic as opposed to earlier (1st half 2020: 41.2%, 2nd half 2020: 46.4%, 1st half 2021: 62.0%, 2nd half 2021: 46.5%, interaction *p* value = 0.0014). Predictors of increased mortality included age, the time of final patient enrolment from 1 January 2020, and the proportion of patients receiving corticosteroids, and reduced duration of ECMO run.

**Conclusions:**

The mortality rate for patients receiving ECMO for COVID-19-related ARDS has increased as the pandemic has progressed. The reasons for this are likely multifactorial; however, as outcomes for these patients evolve, the decision to initiate ECMO should include the best contextual estimate of mortality at the time of ECMO initiation.

**Supplementary Information:**

The online version contains supplementary material available at 10.1186/s13054-022-04011-2.

## Introduction

Extracorporeal membrane oxygenation (ECMO) has been used extensively for coronavirus disease 2019 (COVID-19)-related acute respiratory distress syndrome (ARDS). However, it is highly resource intensive, leading to challenges in provision during the pandemic [1]. A systematic review and meta-analysis examining patients who received ECMO for COVID-19 in 2020 reported a 37% mortality rate [2]. As the pandemic progressed, treatment practices and patterns evolved, and newer variants of severe acute respiratory syndrome coronavirus 2 (SARS-CoV-2) emerged. Alongside these changes, contemporaneous studies reported increasing mortality rates and longer duration of ECMO runs in patients with COVID-19 ARDS. The mortality rate reported by the Extracorporeal Life Support Organisation (ELSO) registry data for the use of ECMO in COVID-19 increased from 37% in early 2020 to 52% by the end of 2020 [3, 4], demonstrating the dynamic nature of clinical outcomes during the course of the pandemic.

While subsequent single-centre studies have shown similar trends, the mortality rates for patients receiving ECMO for COVID-19 appear variable globally, with reports of rates ranging from 17.5% to 68% in the first 18 months of the pandemic [5]. Several reasons related to patient, disease, and treatment factors have been postulated for this and include increased virulence of SARS-CoV-2 variants [5, 6]; changes in patient selection patterns based, at times, on local resource availability; changes in interventions, including the need of using prolonged noninvasive forms of mechanical ventilation and delays in endotracheal intubation due to the overwhelming number of patients with respiratory failure; and the use of immunomodulators such as corticosteroids and interleukin-6 receptor antagonists [3, 7]. Based on this, we performed an updated systematic review and meta-analysis to summarise outcome data during the first 2 years of the pandemic, including the changes in mortality trends, and identify risk factors for unfavourable outcomes in order to guide clinical decision-making and further research.

## Methodology

### Search strategy and selection criteria

We registered the protocol with PROSPERO (CRD42021271202) and conducted the review in adherence with the Preferred Reporting Items for Systematic Reviews and Meta-analyses (PRISMA) Statement (Additional file 1: Table S1) [8]. We searched MEDLINE, Embase, Cochrane and Scopus databases from 1 December 2019 to 26 January 2022 using the following keywords and their variations: “extracorporeal membrane oxygenation”, “extracorporeal life support”, “SARS-CoV-2” and “COVID-19” (Additional file 1: Table S2). We also reviewed the reference lists of included studies and review articles on the topic. We included studies or online registries reporting on at least 10 adult patients with COVID-19 requiring ECMO. We excluded any studies primarily reporting on animals or paediatric patients (< 16 years old). In the case of overlapping patient data, we included the largest study and excluded any other overlapping studies.

### Data collection and risk of bias assessment

We collected data using a prespecified data extraction form. Authors were contacted for additional data where necessary (Additional file 1: Table S3). We assessed individual study risk of bias using the appropriate Joanna Briggs Institute checklist for case series or cohort studies. We assessed certainty of evidence using the Grading of Recommendations, Assessments, Developments and Evaluations (GRADE) approach [9]. The screening of studies, data collection, and risk of bias assessment were conducted independently and in duplicate by RRL and JJLS, and FA assisted with the risk of bias assessment. Conflicts were resolved by consensus or by KR. Where there was missing data, we contacted the corresponding authors of each study to obtain additional data for analysis.

### Data synthesis

The primary outcome was mortality at the longest recorded time of follow-up. Secondary outcomes included ICU and hospital and length of stay, duration of invasive mechanical ventilation, duration of ECMO, and complications during ECMO (which we then classified according to the broad groups described by ELSO). We performed random-effects meta-analyses (DerSimonian and Laird) based on the logit transformation [10–12], and computed 95% confidence intervals (CIs) using the Clopper–Pearson method [13]. As inter-study heterogeneity in observational studies tends to be overestimated by I^2^ statistics, we assessed statistical heterogeneity (inconsistency) as part of the GRADE approach [9], using I-squared but also the Chi-squared test and visual inspection of the forest plots [14]. We assessed for publication bias qualitatively using visual inspection of funnel plots, and quantitatively using Egger’s regression test. We corrected for small-study effects using the random-effects trim-and-fill (R_0_ estimator) procedure. As some centres which published studies on their patient cohort report that patient data to the ELSO registry, there is a risk of duplicating patient data when including studies reporting on data from the ELSO registry. Hence, we conducted a sensitivity analysis excluding any studies reporting on ELSO registry data. We also conducted a second analysis excluding studies with high risks of bias (defined as JBI score < 7) and analysed the mortality among studies specifically reporting on outcomes of patients receiving venovenous ECMO (VV-ECMO). We present survival outcomes as pooled proportions, while continuous outcomes are presented as pooled means, both with corresponding 95% CIs.

We conducted pre-specified subgroup analysis based on the geographical region (North America, Latin America, Asia–Pacific, Europe, Southwest Asia and Africa), as well as by time period (every six months from 1 January 2020, defined by the date of enrolment of the last patient included in each study). We conducted univariable meta-regression when at least 6 data points were reported, to explore potential sources of heterogeneity, or prognostically relevant prespecified study-level covariates (date of last patient enrolment [per 100 days from 1 January 2020], age [per year], proportion of male patients, and patients receiving corticosteroids and interleukin blockers [percentage], body mass index [per 1 kg/m^2^], SOFA score [every increase by 1 point], PaO_2_/FiO_2_ ratio [increase by 1], duration of ECMO cannulation, time from symptoms to mechanical ventilation and time from mechanical ventilation to ECMO [days]). For continuous variables, we pooled the means from the aggregate data presented in each study as per Wan et al. [15]. A *p* value of < 0.05 was defined as statistically significant for our analyses. We performed all statistical analyses using R 4.0.2.

### Post hoc analysis

We investigated the impact of time of last patient enrolment from Jan 1, 2020 on the duration of ECMO, ICU and hospital lengths of stay using study-level meta-regression. In addition, given the disparity in sample sizes, we conducted an exploratory meta-regression of sample size with mortality rates. As studies might recruit patients over a period of time, we conducted a meta-regression of the mean date of patient enrolment (defined as the midpoint between the date of first and last patient enrolment within each study) and mortality. Finally, we conducted an exploratory subgroup analysis based on the duration of follow-up reported by each study.

### Role of the funding source

There was no funding source for this study.

## Results

### Study selection and characteristics

**Fig. 1:**
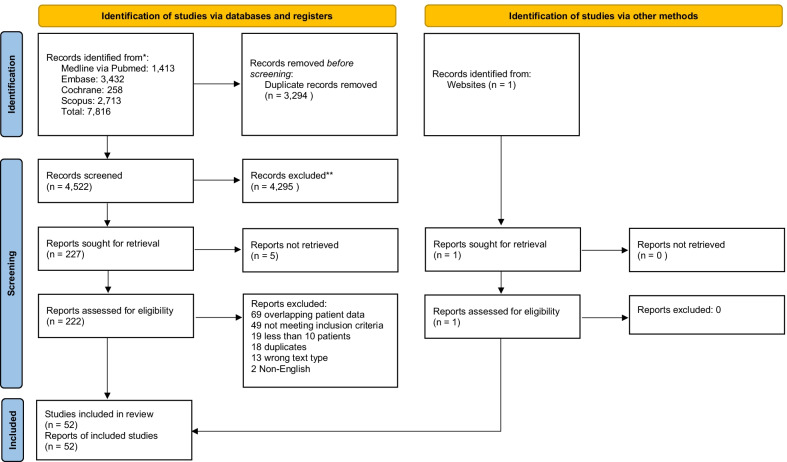
Preferred Reporting Items for Systematic Reviews and Meta-analyses flow diagram

Of 4522 citations, we reviewed 222 full-texts and included 52 studies totalling 18,211 patients receiving ECMO for COVID-19, in the meta-analysis (Fig. 1, Additional file 1: Table S4) [3, 5, 16–65]. All studies were retrospective and observational in nature; 22 studies were conducted by studies centres from Europe [5, 17, 19, 23, 27, 29, 31–34, 36, 39, 42, 43, 45, 46, 50, 55, 56, 60, 63, 65], 17 from North America [16, 18, 20–22, 24, 25, 37, 40, 47, 48, 52–54, 57, 58, 61], 6 from the Asia–Pacific [26, 28, 35, 38, 41, 44], 2 from South-West Asia and Africa,[51, 62] 2 from Latin America [30, 64], and 3 were studies conducted by centres from multiple ELSO regions [3, 49, 59], of which 2 were based on registry data [3, 49]. The pooled age was 52.5 years (95% CI 50.7 to 54.3), and the majority of patients were male (75.0%, 95% CI 72.4% to 77.4%) and obese (BMI: 31.0, 95% CI 30.2 to 31.8). The pooled PaO_2_/FiO_2_ ratio at the time of ECMO initiation was 72.4 (95% CI 68.8 to 76.0), and the pooled sequential organ failure assessment (SOFA) score was 9.24 (95% CI 8.27 to 10.23). The time from onset of clinical symptoms or hospitalisation to initiation of invasive mechanical ventilation was 7.3 days (95% CI 4.1 to 10.5), and ECMO cannulation occurred after an additional 4.89 days (95% CI 4.26 to 5.53) of invasive mechanical ventilation.

From 43 studies, 13,422 of 14,022 patients (95.9%, 95% CI 94.2% to 97.1%) were supported with venovenous (VV)-ECMO. Of the remaining patients, 489 (3.5%) patients were supported with veno-arterial (VA)-ECMO, 97 (0.7%) patients were supported with veno-veno-arterial or veno-arterio-venous ECMO, and 14 (0.1%) patients converted from VV-ECMO to another form of ECMO. The study characteristics, patient demographics, and patient outcomes are summarised in Additional file 1: Table S4a, while the pre-ECMO ventilatory parameters are tabulated in Additional file 1: Table S4b. The intra-study risk of bias is summarised in Additional file 1: Table S5, while the GRADE assessment can be found in Additional file 1: Table S6. Most studies were of good quality, scoring > 7 on the appropriate JBI checklist.

### Primary meta-analysis

**Fig. 2:**
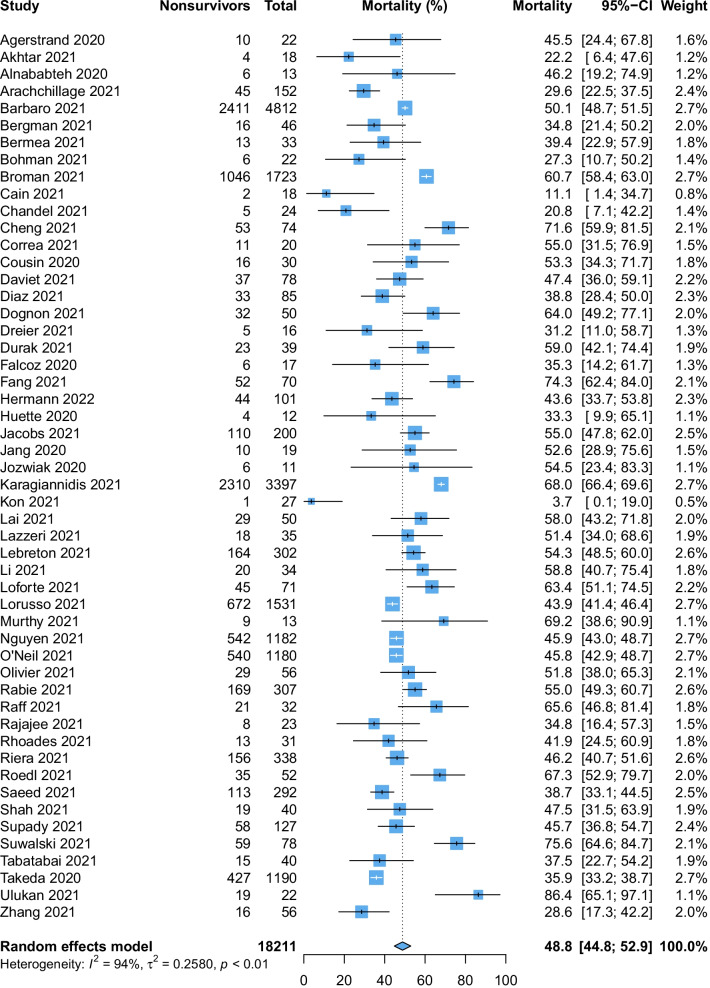
Forest plot demonstrating the pooled mortality among patients receiving extracorporeal membrane oxygenation for coronavirus disease 2019

**Fig. 3:**
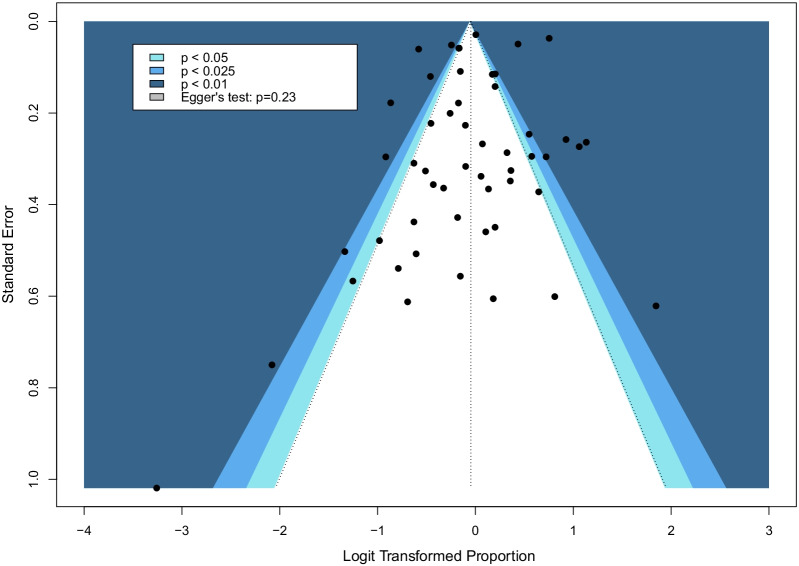
Funnel plot post random-effects trim-and-fill (R_0_ estimator) analysis. Bubbles that are black in fill represent the studies included in the meta-analysis, bubbles that are hollow represent filled-in studies based on the trim-and-fill estimator

The pooled mortality rate among patients requiring ECMO for COVID-19 was 48.8% (95% CI 44.8% to 52.9, high certainty evidence, p_egger_: 0.23, Fig. 2). Sensitivity analysis excluding studies with high risk of bias (48 studies, 14,884 patients, 48.8%, 95% CI 44.4% to 53.2%) and ELSO registry data (48 studies, 8965 patients, 48.6%, 95% CI 44.1% to 53.1%) were consistent with the overall results and conclusions. Sensitivity analysis limited to patients receiving VV-ECMO was also similar with mortality of 47.1% (95% CI 42.2% to 52.1%, Additional file 1: Fig. S1). Mortality after correction of small-study effects using the random-effects trim-and-fill analysis (R_0_ estimator) was 52.5% (95% CI 47.9% to 57.0%, Fig. 3).

### Subgroup analysis

**Fig. 4:**
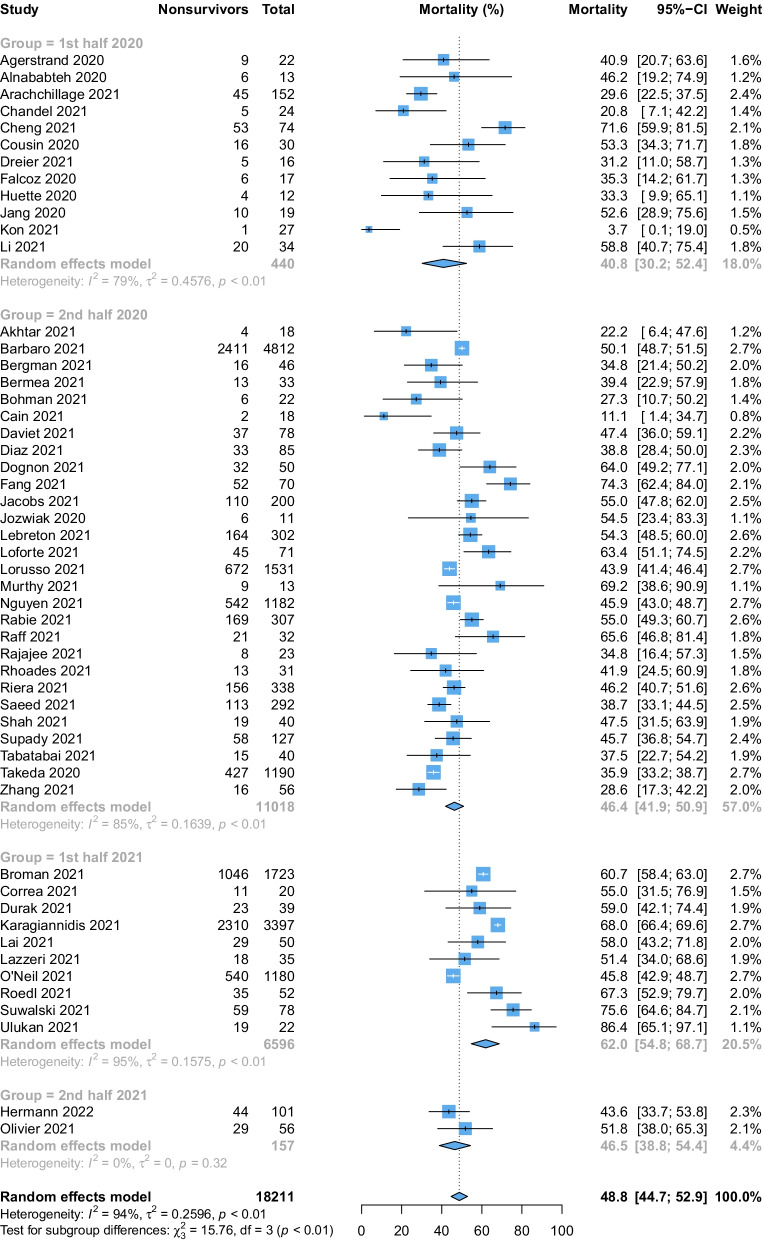
Forest plot demonstrating the pooled mortality among patients receiving extracorporeal membrane oxygenation for coronavirus disease 2019 stratified by time period based on the date of final patient enrolment

**Fig. 5:**
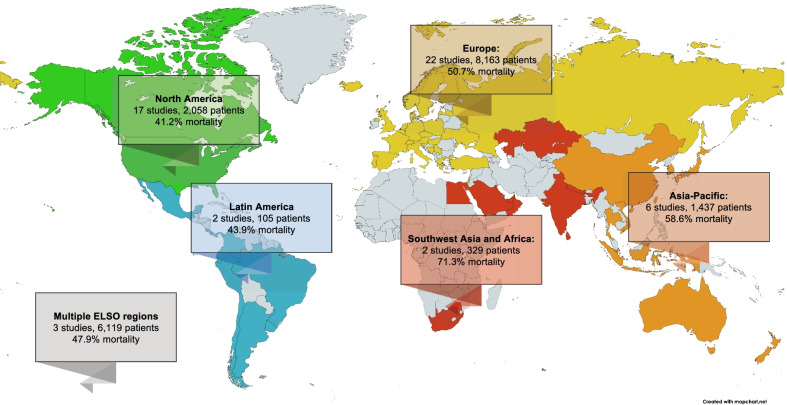
World map demonstrating number of studies, patients, and pooled outcomes for each ELSO region reporting on mortality of extracorporeal membrane oxygenation for coronavirus disease 2019

There was a significant difference in mortality based on the timing of last patient enrolment (interaction *p* value = 0.0014, Fig. 4). Patients enrolled during 2020 had a comparatively lower mortality rate (1st half: 41.2%; 2nd half: 46.4%) than those enrolled in the 1st half (62.0%) and 2nd half of 2021 (46.5%). However, mortality was not different across regions (interaction *p* value = 0.096, Fig. 5 and Additional file 1: Fig. S2). Studies from South West Asia and Africa (71.3%) reported the highest mortality rates, followed by studies from the Asia–Pacific regions (58.6%) and Europe (50.7%). Finally, relatively lower mortality rates were reported by studies from North America (41.2%), Latin America (43.9%) and those across multiple ELSO regions (47.9%). Details of the subgroup analyses are summarised in Additional file 1: Table S7.

### Meta-regression analysis

**Fig. 6:**
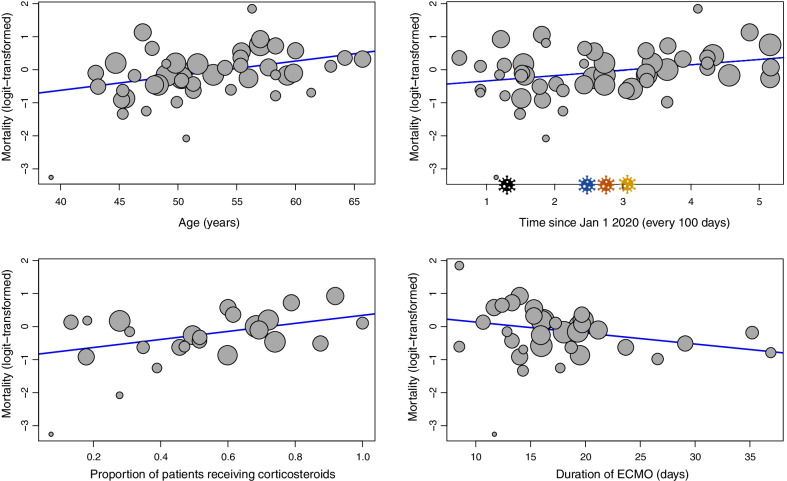
Meta-regression plot demonstrating the association of age, the date of final patient enrolment, the proportion of patients receiving corticosteroids, and the duration of extracorporeal membrane oxygenation, with mortality. Bubble sizes are inverse-variance weighted, and correspond to the variances of each study, i.e. as the variance decreases, bubble size increases. In the meta-regression of mortality and time (top right), the virus icons refer to new SARS-CoV-2 variants - Black: Beta (May 2020); Blue: Alpha (Sep 2020); Orange: Delta (Oct 2020); Yellow: Omicron (Nov 2020)

**Fig. 7.**
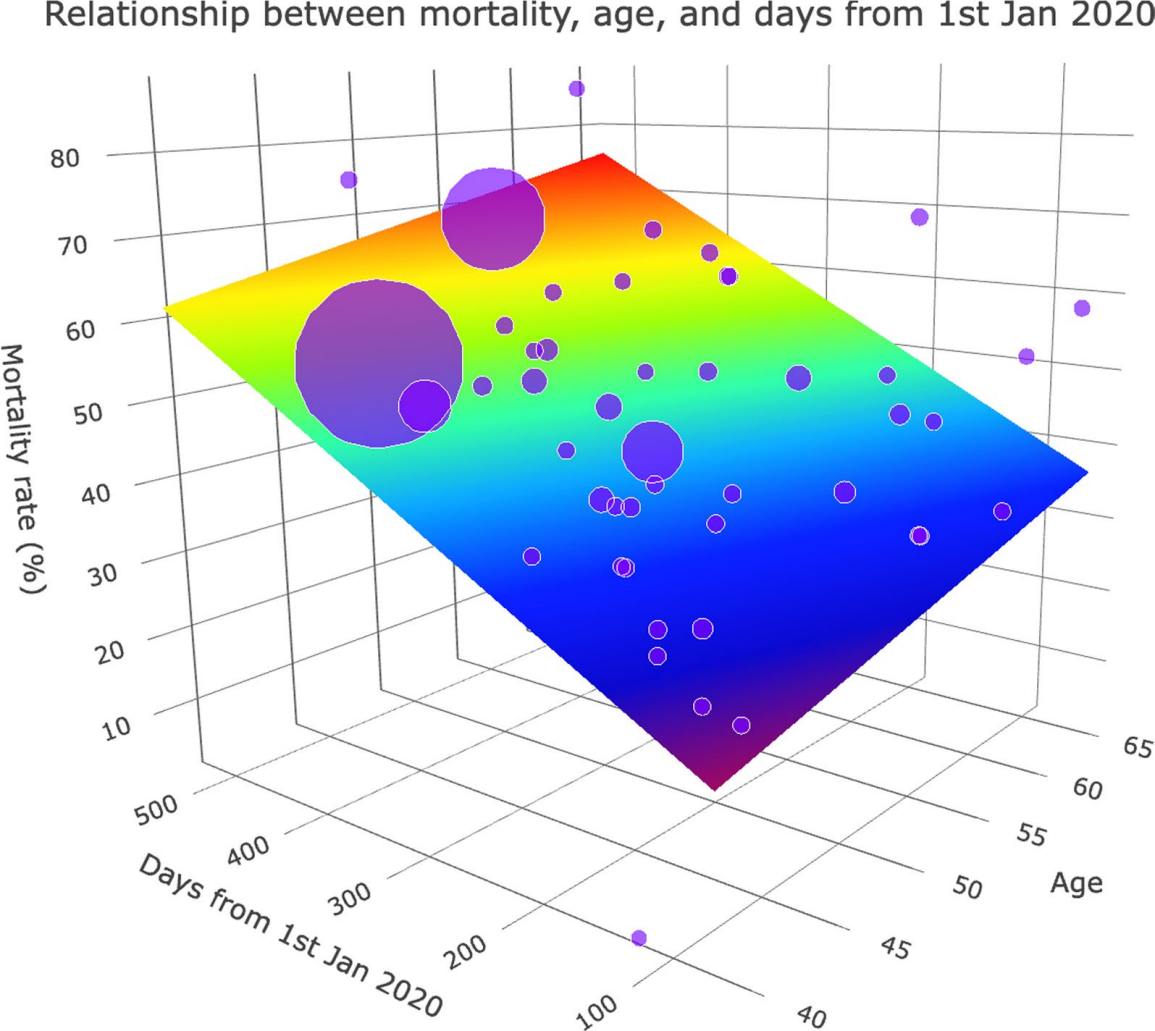
Three-dimensional linear plot demonstrating the association between age, date of final patient enrolment, and mortality. Bubble sizes are inverse-variance weighted and correspond to the variances of each study, i.e. as the variance decreases, bubble size increases. The 3-dimensional sheet follows a rainbow palette: dark red represents a higher mortality rate, while dark blue represents a lower mortality rate

Univariable meta-regression (Figs. 6 and 7) found that the date of last patient enrolment (regression coefficient [B] for every 100 days from 1 January 2020: + 0.16, 95% CI + 0.04 to + 0.29, *p* = 0.012) was associated with increased mortality. Patient factors including age (B: + 0.04, 95% CI + 0.02 to + 0.07, *p* = 0.0014), and the proportion of patients receiving corticosteroids (B: + 1.22, 95% CI + 0.25 to + 2.19, *p* = 0.014) was also associated with increased mortality. However, the duration of ECMO was inversely associated with mortality (B: − 0.03, 95% CI − 0.07 to − 0.0001, *p* = 0.049). Other factors such as body mass index, proportion of male patients, SOFA score, PaO_2_/FiO_2_ ratio, proportion of patients receiving other immunomodulators, duration from clinical symptoms to mechanical ventilation, and duration between mechanical ventilation to ECMO were not associated with mortality. Details of the meta-regression are summarised in Additional file 1: Table S7.

### Secondary outcomes

On average, patients received ECMO for 16.4 days (95% CI 14.9 to 17.9, 35 studies, moderate certainty). The length of ICU stay was 33.5 days (95% CI 29.4 to 37.6, 14 studies, moderate certainty), and the length of hospital stay was 39.2 days (95% CI 33.0 to 45.5, 15 studies, moderate certainty). A total of 10,249 ECMO complications were reported among 37 studies; from 10 studies (5360 patients), 45.7% (95% CI 26.7% to 65.4%) of patients experienced at least one complication while receiving ECMO. The secondary outcomes are presented in Additional file 1: Figs. S3 to S5, and complications are tabulated in Additional file 1: Table S8.

### Post hoc analysis

We conducted a post hoc study-level meta-regression, which demonstrated that the time of last patient enrolment from 1 January 2020 was associated with a longer hospital stay (15 studies, B: + 7.59, 95% CI + 1.51 to + 13.7, *p* = 0.014), but was not significantly associated with the duration of ECMO (35 studies, B: + 0.74, 95% CI − 0.43 to + 1.91, *p* = 0.22) or ICU length of stay (14 studies, B: 2.02, 95% CI − 0.89 to 4.93, *p* = 0.17, Additional file 1: Fig. S6). Sample size was not significantly associated with mortality (52 studies, B: 0.00, 95% CI − 0.0001 to 0.0001, *p* = 0.93). The average date of patient enrolment (defined as the midpoint between the first and last date of patient enrolment within each study) was significantly associated with mortality (52 studies, B: 0.29, 95% CI 0.04 to 0.54 *p* = 0.021, Additional file 1: Fig. S7). 30 studies (8240 patients) reported the duration of follow-up. In-hospital mortality was reported in 15 studies (50.6%, 95% CI 43.4% to 57.8%), and ICU mortality (57.0%, 95% CI 46.1% to 67.2%) and 90-day mortality (52.3%, 95% CI 44.7% to 59.8%) were reported by 4 studies each. 60-day and 180-day mortality were reported in 2 studies, and 30-day, 70-day and 120-day mortality were reported in 1 study each. Additional file 1: Figure S8 summarises the details of this subgroup analysis.

## Discussion

This systematic review and meta-analysis reported a pooled mortality rate of 48.8% (95% CI 44.8% to 52.9%) among patients receiving ECMO for COVID-19, which were robust in a number of sensitivity analyses. Mortality was positively associated with age, time of last patient enrolment from 1 January 2020, and the proportion of patients receiving corticosteroids, while mortality was negatively associated with ECMO duration. The pooled ECMO duration was approximately 16 days, and patients remained in the ICU for 33.5 days, and in the hospital for 39 days.

Consistent with previous analyses, this review found that age was associated with increased mortality [3]. An important evolution in the management of severe COVID-19 was the use of corticosteroids and interleukin-6 receptor (IL-6R) antagonists [66]. While corticosteroids reduce mortality in COVID-19 [67], some studies have suggested that there exist steroid-responsive and -resistant phenotypes [68]. It is possible that a subgroup of patients receiving this treatment, who would otherwise progress to severe ARDS, eventually improved and did not require ECMO. As such, the increase in mortality might stem from selection bias for patients with more severe ARDS refractory to adjunctive therapies than earlier on in the pandemic. Even amongst those who eventually require ECMO, a study of 40 patients found that mortality rates of patients receiving ECMO after a full 10-day course of dexamethasone was 100% compared to 57% where ECMO was instituted before completing the course of dexamethasone [69]. In addition to this, immunomodulatory treatment might be associated with increased rates of secondary infections, which itself is associated with increased mortality rates [70], though this is not confirmed by all the available evidence [71]. In addition, other possible factors that might also confound patient selection longitudinally include the evolution of the SARS-CoV-2 virus, the more common and prolonged use of noninvasive ventilation, and changes in patient selection based on local resource availability changes. Interestingly, a longer duration of ECMO was associated with reduced mortality. This has previously been described and is partially attributable to immortal time bias—patients need to survive a certain duration of time while supported with ECMO to fulfil the criteria for weaning, while patients who had early life-threatening complications might have had their ECMO stopped earlier for futility or died [2, 72]. Another possible factor to consider is the potential conversion of ECMO as a bridge to recovery to a bridge to lung transplant. This siphons off some of the sickest patients who have the longest ECMO runs and would not have survived without ECMO and the lung transplant. This could have skewed the data, resulting in an increased mean duration of ECMO reported at the study level.

An individual participant data meta-analysis of randomised controlled trials (RCTs) investigating ECMO in ARDS showed that ECMO can significantly reduce mortality in a well-selected and defined population [73]. Prior to 2021, observational studies reported that the mortality of patients receiving ECMO for COVID-19-related ARDS was similar to those enrolled in these prior RCTs [2]. Yet, the rise in mortality raised concerns regarding the role of ECMO as a management strategy for COVID-19-related ARDS as the pandemic progressed. It is difficult to ascertain to what extent the temporal increase in mortality is an evolving outcome with respect to COVID-19. This is further compounded by the challenges in determining the mortality benefit conferred by ECMO in the absence of randomised controlled trials (RCTs), which have their own inherent challenges in the context of ECMO and the pandemic [74–76]. Nonetheless, our analysis of study-level data supports the hypothesis that younger patients, and those with shorter durations of mechanical ventilation prior to ECMO are more likely to benefit, as elucidated by previous studies in and outside of COVID-19 ARDS [77, 78]. Finally, decision-making regarding ECMO candidacy should evolve alongside these changing outcomes [7, 79].

This study has important strengths. First, this meta-analysis of more than 18,000 patients summarises the largest and most comprehensive cohort of patients requiring ECMO for COVID-19 to date. While previous reviews were limited by the number of studies [80], our analysis is with a larger sample size, allows for more precision in the pooled estimate, and allows us to more clearly elicit factors that are associated with mortality. In addition to being concordant with previous studies [3, 5], our study provides confirmation of the increase in mortality from a much larger sample size and from multiple studies throughout the world. In addition, we included data reported by registries and studies which were not captured by the ELSO registry. Second, the use of subgroup and meta-regression analyses allowed us to account for certain factors which might have contributed to the heterogeneity of the pooled estimate. Third, we carried out careful risk of bias evaluation of the included studies and used the GRADE approach to assess the certainty of evidence. There are, nonetheless, several limitations which we recognise. First, there is a risk of overlapping patient data as some centres which published studies on their patient cohort report that patient data to the ELSO registry. We mitigated this via a sensitivity analysis excluding ELSO registry reviews, which showed that the pooled estimate remained very similar. Second, the variability in systems of care and indications of ECMO for COVID-19, in the lack of adjustment methods for confounders, resulted in significant heterogeneity of the pooled estimate. While this may partially be accounted for using subgroup and meta-regression analyses, our analyses are limited by study-level data which does not allow us to investigate associations at the patient level, or longitudinally over time. In addition, not all the data are described by all the included studies. In situations where very few studies reported on a covariate for subgroup or meta-regression analysis, the analysis is limited in terms of generalisability and power. Third, the limited sample size of studies included in the second half of 2021 (157 patients) is not sufficient to draw any conclusions about the mortality rates during this time period. Finally, our meta-analysis is only applicable to current practices and is based on patients who were enrolled predominantly through the first half of 2021. Much remains to be known about the long-term impact of COVID-19 and ECMO in these patients [81, 82]. As such, the findings of this review need to be interpreted in context and clinical practice may evolve further.

## Conclusions

In conclusion, our review summarising the updated literature on the use of ECMO for COVID-19 demonstrated an increase in mortality in 2021, likely due to a combination of demographic, disease, and intervention factors. It is evident that a one-size fits all protocolised approach to ECMO, used earlier in the pandemic, may not be as applicable as newer variants emerge, clinical patterns vary and management for severe COVID-19 changes. Despite the increase in mortality over time, ECMO still serves an important role as supportive therapy for select patients. Physicians should carefully weigh the potential benefits and harms of ECMO for each patient in the context of resource availability, the individual’s disease course, and local experience and mortality rates in order to decide on ECMO candidacy [7].

## Supplementary Information

**Additional file 1**. Supplementary Appendix (Figures S1 to S8, Tables S1 to S8).

## Data Availability

All data generated or analysed during this study are included in the published studies and their supplementary information files.

## Abbreviations

ARDS: Acute respiratory distress syndrome
COVID-19: Coronavirus disease 2019
ECMO: Extracorporeal membrane oxygenation
ELSO: Extracorporeal Life Support Organisation
GRADE: Grading of Recommendations, Assessment, Development and Evaluations
ICU: Intensive care unit
IL-6R: Interleukin-6 receptor
PRISMA: Preferred Reporting Items for Systematic Reviews and Meta-analyses
PROSPERO: International Prospective Register of Systematic Reviews
SARS-CoV-2: Severe acute respiratory syndrome coronavirus 2
SOFA: Sequential organ failure assessment
VA: Venoarterial
VV: Venovenous
