# Supplementary material for: Evolving outcomes of extracorporeal membrane oxygenation during the first 2 years of the COVID-19 pandemic: a systematic review and meta-analysis
Source: Crit Care. 2022 May 23;26:147. doi: 10.1186/s13054-022-04011-2 (PMC9125014; doi:10.1186/s13054-022-04011-2)
Supplement: Supplementary file 1 — Additional file 1. Supplementary Appendix (Figures S1 to S8, Tables S1 to S8). [file 13054_2022_4011_MOESM1_ESM.pdf]

Extracorporeal membrane oxygenation for coronavirus disease 2019: an updated systematic review and meta-analysis

Supplementary appendix

*Ryan Ruiyang Ling,
*Kollengode Ramanathan, MD,
Jackie Jia Lin Sim,
Suei Nee Wong, MSc,
Ying Chen, PhD,
Faizan Amin, MD,
Shannon M. Fernando, MD,
Bram Rochwerg, MD,
Eddy Fan, PhD,
Ryan P Barbaro, MD,
Graeme MacLaren MSc,
†Kiran Shekar, PhD,
†Prof Daniel Brodie, MD.

---

*Joint first authors
†Joint senior authors

Corresponding author
Kollengode Ramanathan,
Cardiothoracic Intensive Care Unit, National University Heart Centre, National University Hospital,
Level 9, 1E Kent Ridge Road, Singapore 119228
Email: surrkr@nus.edu.sg

Table of Contents

Figure S1. Pooled mortality among patients receiving venovenous extracorporeal membrane oxygenation for coronavirus disease 2019. 3

Figure S2. Pooled mortality among patients receiving extracorporeal membrane oxygenation for coronavirus disease 2019 stratified by region. 4

Figure S3. Pooled duration of ECMO support 5

Figure S4. Pooled intensive care unit length of stay amongst patients receiving extracorporeal membrane oxygenation for coronavirus disease 2019 5

Figure S5. Pooled hospital length of stay among patients receiving extracorporeal membrane oxygenation for coronavirus disease 2019 6

Figure S6. Association between time of last patient enrolment since 1 January 2020 (per 100 days) with duration of extracorporeal membrane oxygenation support, intensive care unit length of stay, and hospital length of stay. 7

Figure S7. Association between average time patient enrolment since 1 January 2020 (per 100 days, defined as midpoint between date of first and last patient enrolment) and mortality. 8

Figure S8. Post-hoc subgroup analysis of mortality for patients receiving extracorporeal membrane oxygenation for coronavirus disease 2019, stratified by duration of follow up. 9

Table S1. Preferred Reporting Items for Systematic reviews and Meta-Analyses (PRISMA) 2020 Checklist 10

Table S3. Data collection template 15

Table S4a. Demographics and outcomes of included studies 16

Table S4b. Pre-ECMO ventilatory parameters 19

Table S5. Joanna Briggs Institute Checklist 21

Table S6. Grading of Recommendations, Assessment, Development, and Evaluations 23

Table S7. Summary of subgroup and meta-regression analysis 24

Table S8: Complications reported by included studies 25

Figure S1. Pooled mortality among patients receiving venovenous extracorporeal membrane oxygenation for coronavirus disease 2019.

Study	Nonsurvivors	VV–ECMO	Mortality	95%–CI	Weight
Agerstrand 2020	8	15	53.3	[26.6; 78.7]	1.7%
Akhtar 2021	4	16	25.0	[7.3; 52.4]	1.7%
Alnababteh 2020	6	13	46.2	[19.2; 74.9]	1.6%
Arachchillage 2021	45	152	29.6	[22.5; 37.5]	2.8%
Barbaro 2021	2179	4492	48.5	[47.0; 50.0]	3.0%
Bergman 2021	16	46	34.8	[21.4; 50.2]	2.3%
Bermea 2021	13	33	39.4	[22.9; 57.9]	2.2%
Bohman 2021	6	22	27.3	[10.7; 50.2]	1.9%
Cain 2021	2	18	11.1	[1.4; 34.7]	1.8%
Chandel 2021	5	24	20.8	[7.1; 42.2]	2.0%
Cousin 2020	16	30	53.3	[34.3; 71.7]	2.1%
Daviet 2021	37	77	48.1	[36.5; 59.7]	2.6%
Diaz 2021	31	82	37.8	[27.3; 49.2]	2.6%
Dognon 2021	32	50	64.0	[49.2; 77.1]	2.4%
Dreier 2021	5	16	31.2	[11.0; 58.7]	1.7%
Durak 2021	23	38	60.5	[43.4; 76.0]	2.3%
Fang 2021	52	70	74.3	[62.4; 84.0]	2.5%
Hermann 2022	38	95	40.0	[30.1; 50.6]	2.6%
Huette 2020	4	12	33.3	[9.9; 65.1]	1.5%
Jacobs 2021	101	188	53.7	[46.3; 61.0]	2.8%
Jang 2020	9	16	56.2	[29.9; 80.2]	1.7%
Jozwiak 2020	6	11	54.5	[23.4; 83.3]	1.4%
Karagiannidis 2021	2310	3397	68.0	[66.4; 69.6]	3.0%
Kon 2021	1	27	3.7	[0.1; 19.0]	2.1%
Lai 2021	26	47	55.3	[40.1; 69.8]	2.4%
Lazzeri 2021	18	35	51.4	[34.0; 68.6]	2.2%
Lebreton 2021	155	288	53.8	[47.9; 59.7]	2.9%
Loforte 2021	42	67	62.7	[50.0; 74.2]	2.5%
Murthy 2021	9	13	69.2	[38.6; 90.9]	1.6%
O'Neil 2021	540	1180	45.8	[42.9; 48.7]	2.9%
Olivier 2021	29	56	51.8	[38.0; 65.3]	2.4%
Rabie 2021	152	288	52.8	[46.8; 58.7]	2.9%
Raff 2021	21	32	65.6	[46.8; 81.4]	2.2%
Rajajee 2021	6	20	30.0	[11.9; 54.3]	1.9%
Rhoades 2021	13	31	41.9	[24.5; 60.9]	2.1%
Riera 2021	163	319	51.1	[45.5; 56.7]	2.9%
Roedl 2021	34	51	66.7	[52.1; 79.2]	2.4%
Saeed 2021	109	280	38.9	[33.2; 44.9]	2.8%
Shah 2021	19	40	47.5	[31.5; 63.9]	2.3%
Supady 2021	58	127	45.7	[36.8; 54.7]	2.7%
Suwalski 2021	46	64	71.9	[59.2; 82.4]	2.5%
Tabatabai 2021	15	40	37.5	[22.7; 54.2]	2.3%
Ulukan 2021	14	18	77.8	[52.4; 93.6]	1.8%
Zhang 2021	16	56	28.6	[17.3; 42.2]	2.4%
**Random effects model**		**11992**	**47.1**	**[42.2; 52.1]**	**100.0%**

Heterogeneity: $I^2 = 93\%$, $\tau^2 = 0.0202$, $p < 0.01$

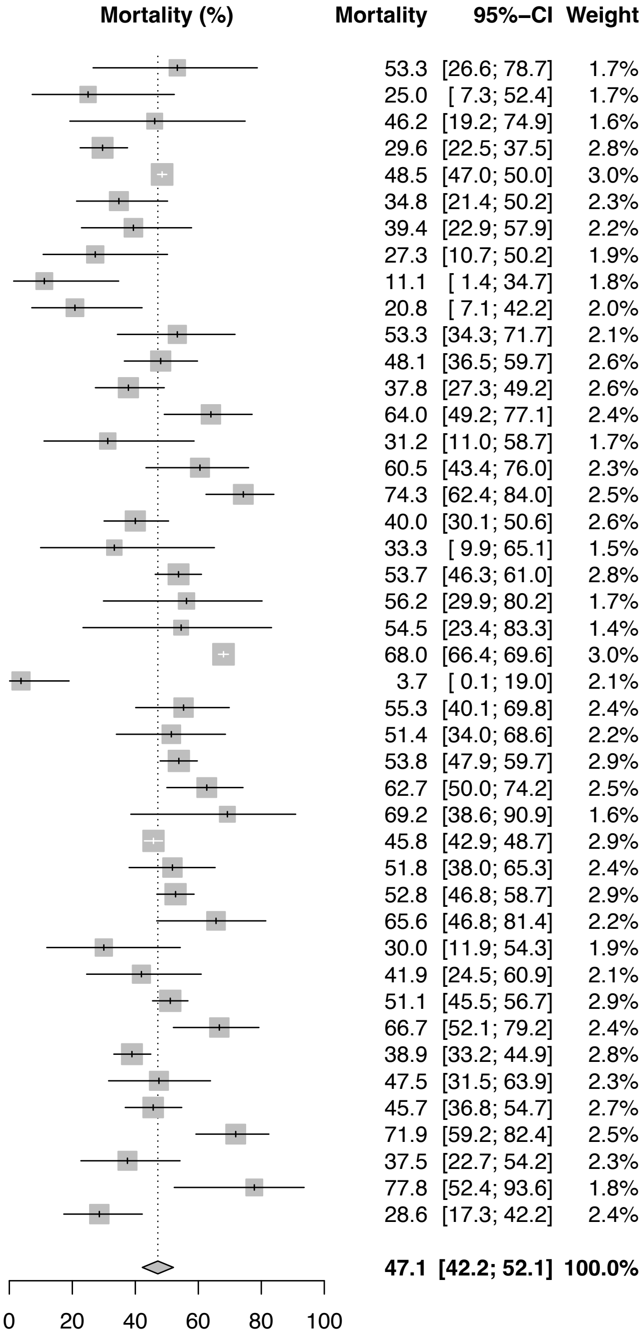

Figure S2. Pooled mortality among patients receiving extracorporeal membrane oxygenation for coronavirus disease 2019 stratified by region.

Study	Nonsurvivors	Total	Mortality	95%−CI	Weight
**Region = North America**					
Agerstrand 2020	10	22	45.5	[24.4; 67.8]	1.6%
Alnababteh 2020	6	13	46.2	[19.2; 74.9]	1.2%
Bergman 2021	16	46	34.8	[21.4; 50.2]	2.0%
Bermea 2021	13	33	39.4	[22.9; 57.9]	1.8%
Bohman 2021	6	22	27.3	[10.7; 50.2]	1.4%
Cain 2021	2	18	11.1	[1.4; 34.7]	0.8%
Chandel 2021	5	24	20.8	[7.1; 42.2]	1.4%
Jacobs 2021	110	200	55.0	[47.8; 62.0]	2.5%
Kon 2021	1	27	3.7	[0.1; 19.0]	0.5%
Murthy 2021	9	13	69.2	[38.6; 90.9]	1.1%
Nguyen 2021	542	1182	45.9	[43.0; 48.7]	2.7%
Raff 2021	21	32	65.6	[46.8; 81.4]	1.8%
Rajajee 2021	8	23	34.8	[16.4; 57.3]	1.5%
Rhoades 2021	13	31	41.9	[24.5; 60.9]	1.8%
Saeed 2021	113	292	38.7	[33.1; 44.5]	2.5%
Shah 2021	19	40	47.5	[31.5; 63.9]	1.9%
Tabatabai 2021	15	40	37.5	[22.7; 54.2]	1.9%
**Random effects model**		**2058**	**41.2**	**[35.2; 47.5]**	**28.5%**
Heterogeneity: $I^2 = 67\%$, $\tau^2 = 0.1486$, $p < 0.01$					
**Region = Europe**					
Akhtar 2021	4	18	22.2	[6.4; 47.6]	1.2%
Arachchillage 2021	45	152	29.6	[22.5; 37.5]	2.4%
Broman 2021	1046	1723	60.7	[58.4; 63.0]	2.7%
Cousin 2020	16	30	53.3	[34.3; 71.7]	1.8%
Daviet 2021	37	78	47.4	[36.0; 59.1]	2.2%
Dognon 2021	32	50	64.0	[49.2; 77.1]	2.0%
Dreier 2021	5	16	31.2	[11.0; 58.7]	1.3%
Durak 2021	23	39	59.0	[42.1; 74.4]	1.9%
Falcoz 2020	6	17	35.3	[14.2; 61.7]	1.3%
Hermann 2022	44	101	43.6	[33.7; 53.8]	2.3%
Huette 2020	4	12	33.3	[9.9; 65.1]	1.1%
Jozwiak 2020	6	11	54.5	[23.4; 83.3]	1.1%
Karagiannidis 2021	2310	3397	68.0	[66.4; 69.6]	2.7%
Lazzeri 2021	18	35	51.4	[34.0; 68.6]	1.9%
Lebreton 2021	164	302	54.3	[48.5; 60.0]	2.6%
Loforte 2021	45	71	63.4	[51.1; 74.5]	2.2%
Lorusso 2021	672	1531	43.9	[41.4; 46.4]	2.7%
Olivier 2021	29	56	51.8	[38.0; 65.3]	2.1%
Riera 2021	156	338	46.2	[40.7; 51.6]	2.6%
Roedl 2021	35	52	67.3	[52.9; 79.7]	2.0%
Suwalski 2021	59	78	75.6	[64.6; 84.7]	2.1%
Zhang 2021	16	56	28.6	[17.3; 42.2]	2.0%
**Random effects model**		**8163**	**50.7**	**[44.5; 56.9]**	**44.1%**
Heterogeneity: $I^2 = 95\%$, $\tau^2 = 0.2650$, $p < 0.01$					
**Region = Multiple ELSO regions**					
Barbaro 2021	2411	4812	50.1	[48.7; 51.5]	2.7%
O'Neil 2021	540	1180	45.8	[42.9; 48.7]	2.7%
Supady 2021	58	127	45.7	[36.8; 54.7]	2.4%
**Random effects model**		**6119**	**47.9**	**[44.3; 51.4]**	**7.7%**
Heterogeneity: $I^2 = 74\%$, $\tau^2 = 0.0099$, $p = 0.02$					
**Region = Asia−Pacific**					
Cheng 2021	53	74	71.6	[59.9; 81.5]	2.1%
Fang 2021	52	70	74.3	[62.4; 84.0]	2.1%
Jang 2020	10	19	52.6	[28.9; 75.6]	1.5%
Lai 2021	29	50	58.0	[43.2; 71.8]	2.0%
Li 2021	20	34	58.8	[40.7; 75.4]	1.8%
Takeda 2020	427	1190	35.9	[33.2; 38.7]	2.7%
**Random effects model**		**1437**	**58.6**	**[45.3; 70.7]**	**12.2%**
Heterogeneity: $I^2 = 93\%$, $\tau^2 = 0.3608$, $p < 0.01$					
**Region = Latin America**					
Correa 2021	11	20	55.0	[31.5; 76.9]	1.5%
Diaz 2021	33	85	38.8	[28.4; 50.0]	2.3%
**Random effects model**		**105**	**43.9**	**[30.1; 58.8]**	**3.8%**
Heterogeneity: $I^2 = 41\%$, $\tau^2 = 0.0890$, $p = 0.19$					
**Region = SWAAC**					
Rabie 2021	169	307	55.0	[49.3; 60.7]	2.6%
Ulukan 2021	19	22	86.4	[65.1; 97.1]	1.1%
**Random effects model**		**329**	**71.3**	**[33.5; 92.5]**	**3.6%**
Heterogeneity: $I^2 = 85\%$, $\tau^2 = 1.1505$, $p < 0.01$					
**Random effects model**		**18211**	**48.8**	**[44.8; 52.9]**	**100.0%**
Heterogeneity: $I^2 = 94\%$, $\tau^2 = 0.2580$, $p < 0.01$					
Test for subgroup differences: $\chi^2_5 = 9.35$, df = 5 ($p = 0.10$)					

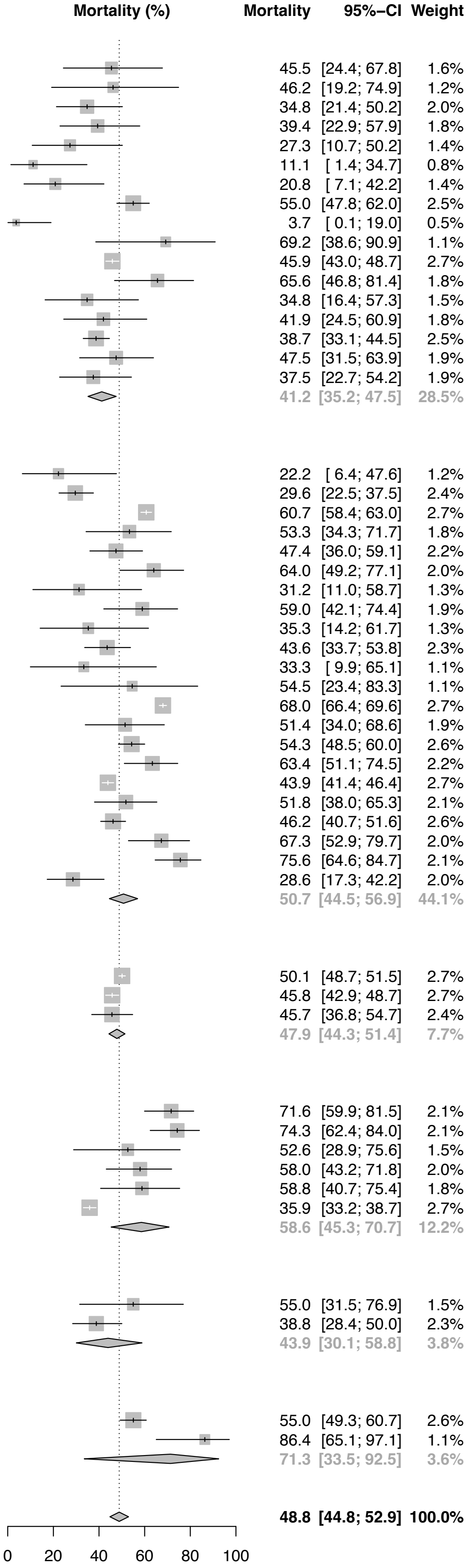

Figure S3. Pooled duration of ECMO support

Study	ECMO	Mean	SD	Duration	95%−CI	Weight
Falcoz 2020	17	8.5	4.4593	8.5	[6.4; 10.6]	3.3%
Huette 2020	12	14.3	10.7086	14.3	[8.3; 20.4]	2.2%
Jang 2020	19	17.3	16.4200	17.3	[9.9; 24.7]	1.9%
Kon 2021	27	11.7	3.1000	11.7	[10.5; 12.9]	3.5%
Alnababteh 2020	13	12.8	6.0360	12.8	[9.6; 16.1]	3.0%
Cheng 2021	74	14.0	9.8200	14.0	[11.8; 16.2]	3.3%
Cousin 2020	30	10.7	5.4489	10.7	[8.7; 12.6]	3.3%
Dreier 2021	16	36.9	29.7000	36.9	[22.3; 51.5]	0.8%
Chandel 2021	24	14.3	9.5000	14.3	[10.5; 18.1]	2.9%
Agerstrand 2020	22	35.2	53.1000	35.2	[13.0; 57.4]	0.4%
Arachchillage 2021	152	19.5	14.2200	19.5	[17.2; 21.8]	3.3%
Lebreton 2021	302	16.0	13.4000	16.0	[14.5; 17.5]	3.4%
Tabatabai 2021	40	29.1	15.9000	29.1	[24.2; 34.0]	2.6%
Zhang 2021	56	14.0	9.9000	14.0	[11.4; 16.6]	3.2%
Bermea 2021	33	13.3	13.9000	13.3	[8.6; 18.0]	2.6%
Akhtar 2021	18	17.7	9.4000	17.7	[13.4; 22.0]	2.7%
Rajajee 2021	23	18.7	19.0000	18.7	[10.9; 26.5]	1.8%
Raff 2021	32	12.4	5.7000	12.4	[10.4; 14.4]	3.3%
Loforte 2021	71	15.3	11.4000	15.3	[12.6; 18.0]	3.2%
Rabie 2021	307	16.2	10.8000	16.2	[15.0; 17.4]	3.4%
Bergman 2021	46	23.7	17.6000	23.7	[18.6; 28.8]	2.5%
Takeda 2020	1190	16.0	13.7800	16.0	[15.2; 16.8]	3.5%
Daviet 2021	78	21.2	15.4900	21.2	[17.7; 24.6]	3.0%
Dognon 2021	50	11.7	6.8700	11.7	[9.8; 13.6]	3.3%
Riera 2021	338	19.3	17.1000	19.3	[17.5; 21.1]	3.4%
Jacobs 2021	200	19.8	16.0833	19.8	[17.6; 22.0]	3.3%
Barbaro 2021	4812	19.5	12.6347	19.5	[19.1; 19.8]	3.5%
Bohman 2021	22	26.6	22.5875	26.6	[17.1; 36.0]	1.5%
Roedl 2021	52	13.3	6.9000	13.3	[11.4; 15.2]	3.3%
Lai 2021	50	15.3	12.2100	15.3	[11.9; 18.7]	3.0%
Ulukan 2021	22	8.5	5.5000	8.5	[6.2; 10.8]	3.3%
Durak 2021	39	19.7	13.8500	19.7	[15.3; 24.0]	2.7%
O'Neil 2021	1180	18.0	14.7596	18.0	[17.2; 18.9]	3.5%
Hermann 2022	101	15.9	10.5300	15.9	[13.9; 18.0]	3.3%
Olivier 2021	56	19.7	16.0000	19.7	[15.5; 23.9]	2.8%
**Random effects model**	**9524**			**16.4**	**[14.9; 17.9]**	**100.0%**

Heterogeneity: $I^2 = 94\%$, $\tau^2 = 16.6745$, $p < 0.01$

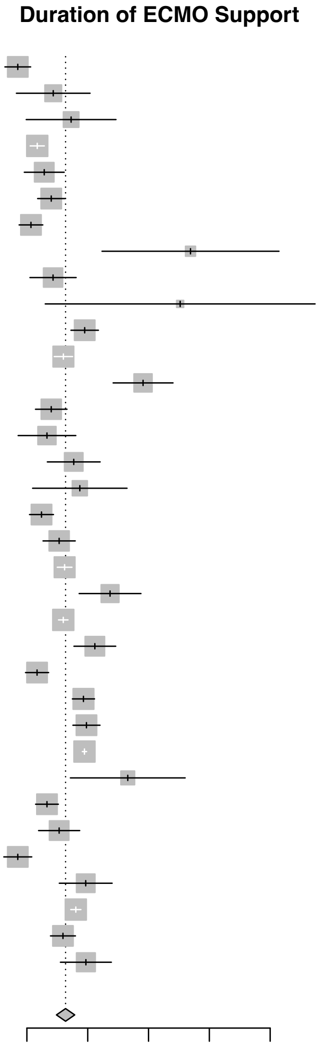

Figure S4. Pooled intensive care unit length of stay amongst patients receiving extracorporeal membrane oxygenation for coronavirus disease 2019

Study	ECMO	Mean	SD	ICULOS	95%−CI	Weight
Cheng 2021	74	22.2	15.3100	22.2	[18.8; 25.7]	8.9%
Dreier 2021	16	73.8	52.0800	73.8	[48.3; 99.4]	2.0%
Lebreton 2021	302	31.3	22.3400	31.3	[28.8; 33.8]	9.2%
Fang 2021	70	29.1	21.8000	29.1	[24.0; 34.2]	8.3%
Cain 2021	18	22.0	21.7000	22.0	[12.0; 32.0]	6.1%
Rajajee 2021	23	41.7	23.7000	41.7	[32.0; 51.4]	6.2%
Diaz 2021	85	39.3	27.1000	39.3	[33.5; 45.1]	8.0%
Nguyen 2021	1182	29.1	17.3000	29.1	[28.1; 30.1]	9.5%
Bergman 2021	46	35.3	22.1900	35.3	[28.9; 41.7]	7.8%
Dognon 2021	50	37.7	30.5300	37.7	[29.2; 46.1]	6.8%
Roedl 2021	52	27.0	23.6000	27.0	[20.6; 33.4]	7.8%
Correa 2021	20	36.3	41.9000	36.3	[17.9; 54.6]	3.3%
Hermann 2022	101	34.2	22.7900	34.2	[29.8; 38.7]	8.6%
Olivier 2021	56	44.7	27.3000	44.7	[37.5; 51.9]	7.4%
**Random effects model**	**2095**			**33.5**	**[29.4; 37.6]**	**100.0%**

Heterogeneity: $I^2 = 84\%$, $\tau^2 = 46.2288$, $p < 0.01$

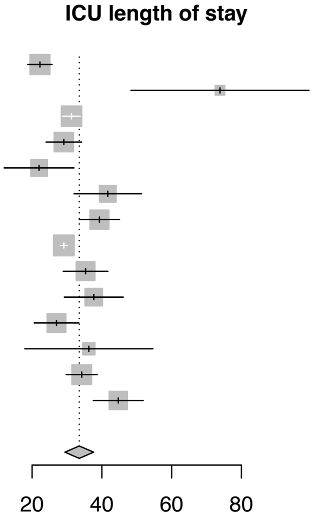

Figure S5. Pooled hospital length of stay among patients receiving extracorporeal membrane oxygenation for coronavirus disease 2019

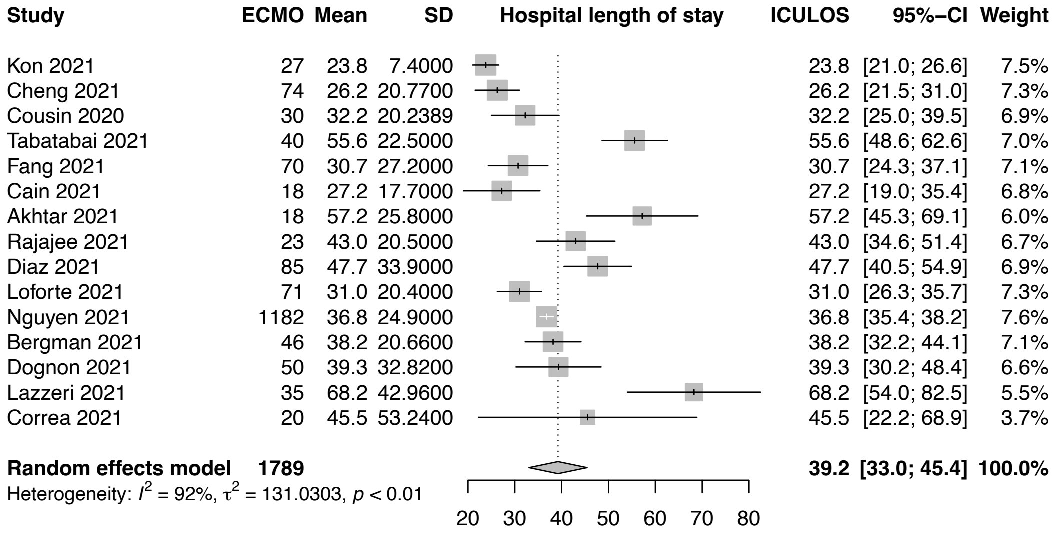

Figure S6. Association between time of last patient enrolment since 1 January 2020 (per 100 days) with duration of extracorporeal membrane oxygenation support, intensive care unit length of stay, and hospital length of stay.

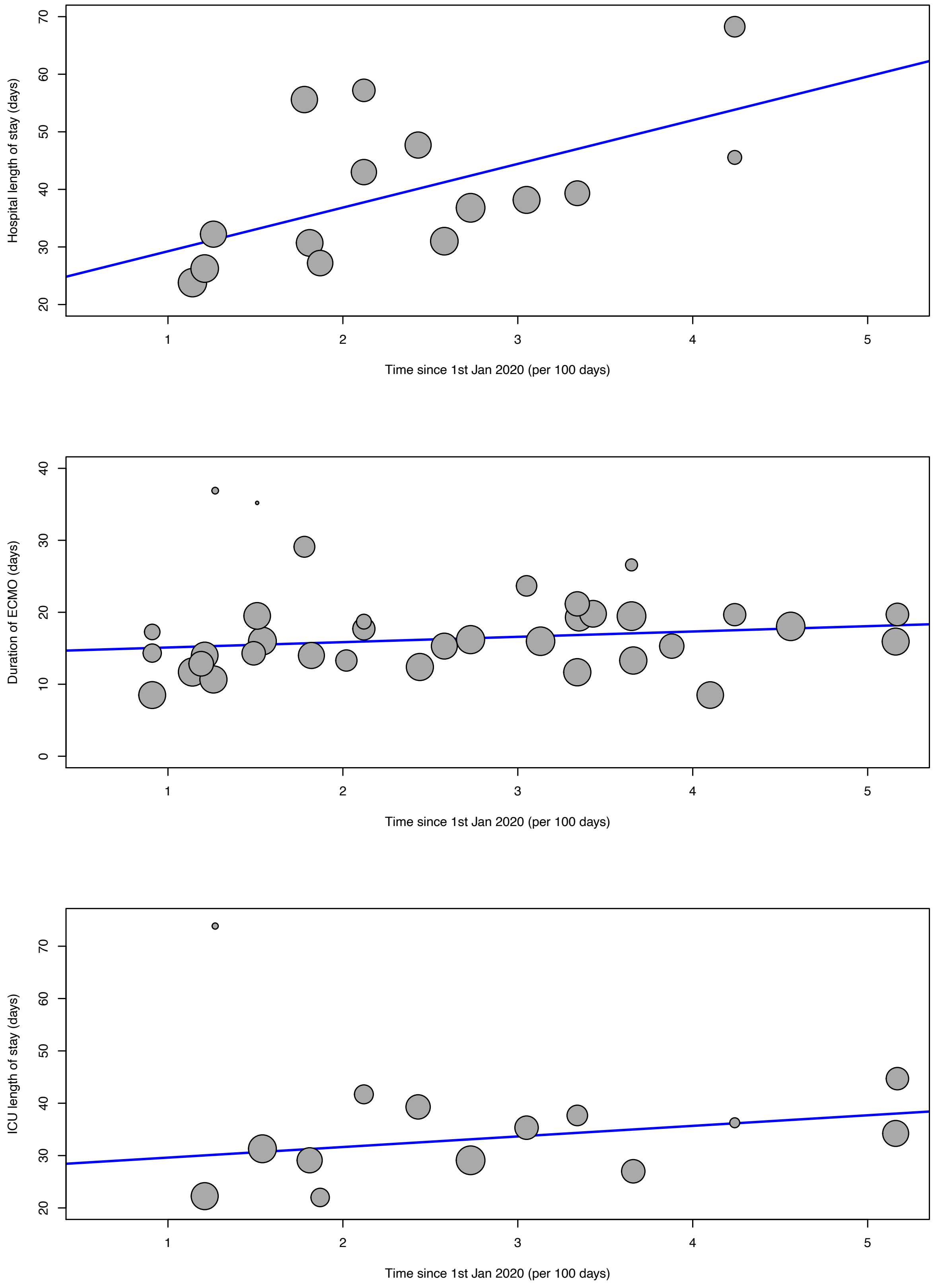

Figure S7. Association between average time patient enrolment since 1 January 2020 (per 100 days, defined as midpoint between date of first and last patient enrolment) and mortality.

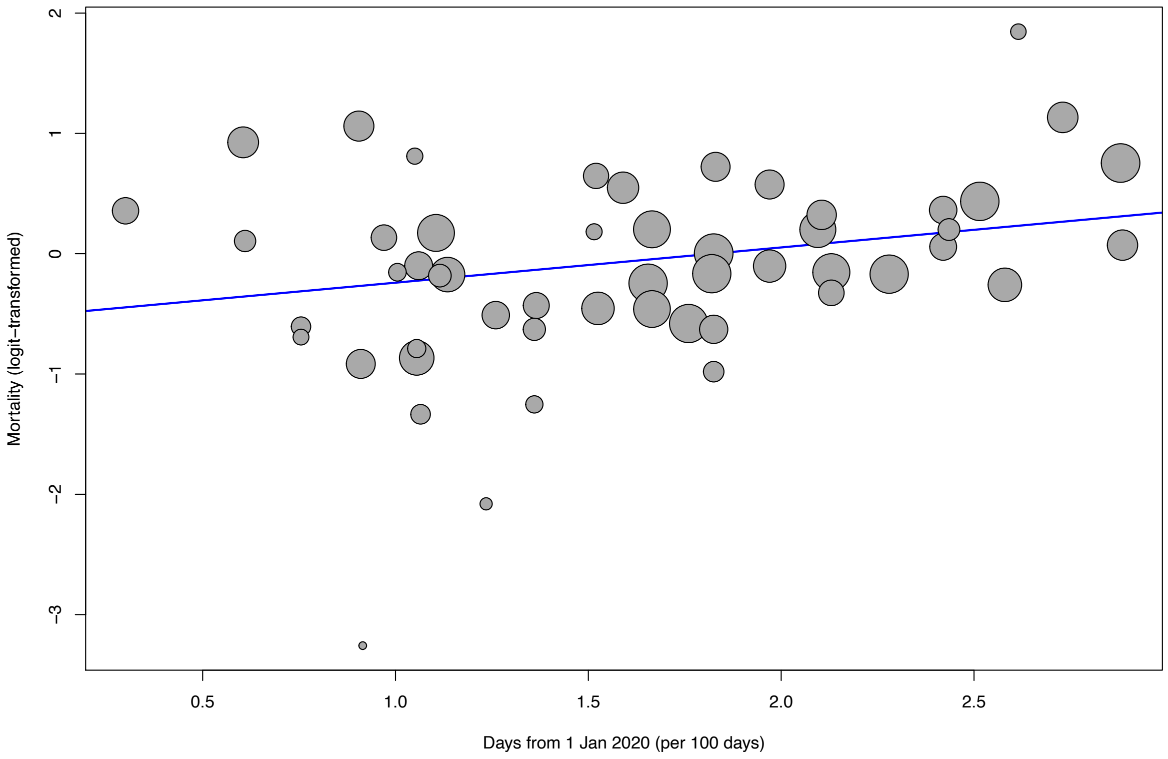

Figure S8. Post-hoc subgroup analysis of mortality for patients receiving extracorporeal membrane oxygenation for coronavirus disease 2019, stratified by duration of follow up.

Study	Nonsurvivors	Total	Mortality	95%−CI	Weight
Days = In−hospital					
Agerstrand 2020	10	22	45.5	[24.4; 67.8]	2.7%
Cain 2021	2	18	11.1	[1.4; 34.7]	1.4%
Cheng 2021	53	74	71.6	[59.9; 81.5]	3.6%
Hermann 2022	44	101	43.6	[33.7; 53.8]	4.0%
Loforte 2021	45	71	63.4	[51.1; 74.5]	3.7%
Murthy 2021	9	13	69.2	[38.6; 90.9]	1.9%
O'Neil 2021	540	1180	45.8	[42.9; 48.7]	4.6%
Olivier 2021	29	56	51.8	[38.0; 65.3]	3.6%
Rajajee 2021	8	23	34.8	[16.4; 57.3]	2.6%
Rhoades 2021	13	31	41.9	[24.5; 60.9]	3.0%
Riera 2021	156	338	46.2	[40.7; 51.6]	4.4%
Saeed 2021	113	292	38.7	[33.1; 44.5]	4.4%
Suwalski 2021	59	78	75.6	[64.6; 84.7]	3.6%
Barbaro 2021	2411	4812	50.1	[48.7; 51.5]	4.6%
Cousin 2020	16	30	53.3	[34.3; 71.7]	3.0%
Random effects model		7139	50.6	[43.4; 57.8]	51.1%
Heterogeneity: $I^2 = 81\%$, $\tau^2 = 0.2399$, $p < 0.01$					
Days = ICU					
Alnababteh 2020	6	13	46.2	[19.2; 74.9]	2.0%
Jang 2020	10	19	52.6	[28.9; 75.6]	2.5%
Lazzeri 2021	18	35	51.4	[34.0; 68.6]	3.2%
Roedl 2021	35	52	67.3	[52.9; 79.7]	3.4%
Random effects model		119	57.0	[46.1; 67.2]	11.1%
Heterogeneity: $I^2 = 12\%$, $\tau^2 = 0.0477$, $p = 0.33$					
Days = 90−day					
Bermea 2021	13	33	39.4	[22.9; 57.9]	3.1%
Daviet 2021	37	78	47.4	[36.0; 59.1]	3.8%
Dognon 2021	32	50	64.0	[49.2; 77.1]	3.4%
Lebreton 2021	164	302	54.3	[48.5; 60.0]	4.4%
Random effects model		463	52.3	[44.7; 59.8]	14.7%
Heterogeneity: $I^2 = 50\%$, $\tau^2 = 0.0438$, $p = 0.11$					
Days = 70−day					
Huette 2020	4	12	33.3	[9.9; 65.1]	1.8%
Random effects model		12	33.3	[13.1; 62.4]	1.8%
Heterogeneity: not applicable					
Days = 60−day					
Falcoz 2020	6	17	35.3	[14.2; 61.7]	2.3%
Zhang 2021	16	56	28.6	[17.3; 42.2]	3.4%
Random effects model		73	30.2	[20.8; 41.7]	5.7%
Heterogeneity: $I^2 = 0\%$, $\tau^2 = 0$, $p = 0.60$					
Days = 30−day					
Supady 2021	58	127	45.7	[36.8; 54.7]	4.1%
Random effects model		127	45.7	[37.2; 54.4]	4.1%
Heterogeneity: not applicable					
Days = 180−day					
Arachchillage 2021	45	152	29.6	[22.5; 37.5]	4.1%
Diaz 2021	33	85	38.8	[28.4; 50.0]	3.9%
Random effects model		237	33.6	[25.3; 43.0]	8.0%
Heterogeneity: $I^2 = 52\%$, $\tau^2 = 0.0441$, $p = 0.15$					
Days = 120−day					
Fang 2021	52	70	74.3	[62.4; 84.0]	3.6%
Random effects model		70	74.3	[62.8; 83.2]	3.6%
Heterogeneity: not applicable					
**Random effects model**		**8240**	**49.2**	**[44.0; 54.4]**	**100.0%**
Heterogeneity: $I^2 = 80\%$, $\tau^2 = 0.2457$, $p < 0.01$					
Test for subgroup differences: $\chi^2_7 = 40.41$, df = 7 ($p < 0.01$)					

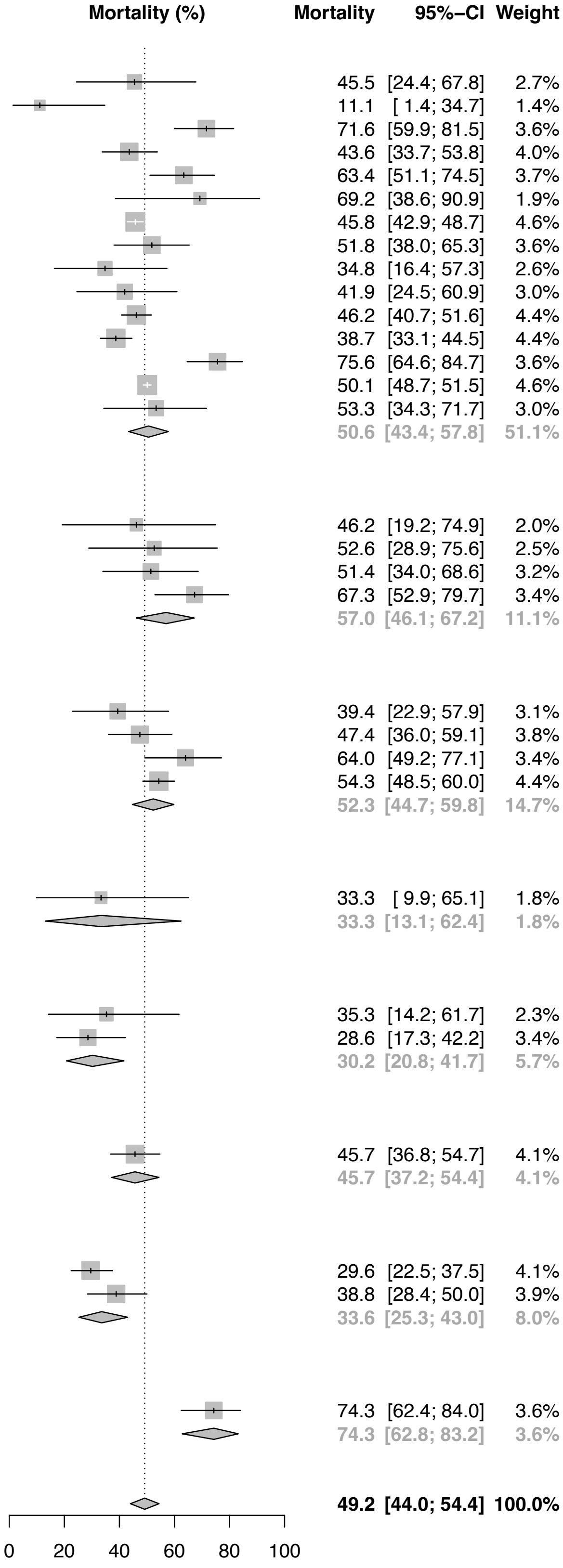

Table S1. Preferred Reporting Items for Systematic reviews and Meta-Analyses (PRISMA) 2020 Checklist

Section and Topic	Item #	Checklist item	Location where item is reported
**TITLE**			
Title	1	Identify the report as a systematic review.	1
**ABSTRACT**			
Abstract	2	See the PRISMA 2020 for Abstracts checklist.	3
**INTRODUCTION**			
Rationale	3	Describe the rationale for the review in the context of existing knowledge.	5
Objectives	4	Provide an explicit statement of the objective(s) or question(s) the review addresses.	5
**METHODS**			
Eligibility criteria	5	Specify the inclusion and exclusion criteria for the review and how studies were grouped for the syntheses.	6
Information sources	6	Specify all databases, registers, websites, organisations, reference lists and other sources searched or consulted to identify studies. Specify the date when each source was last searched or consulted.	5
Search strategy	7	Present the full search strategies for all databases, registers and websites, including any filters and limits used.	Table S2
Selection process	8	Specify the methods used to decide whether a study met the inclusion criteria of the review, including how many reviewers screened each record and each report retrieved, whether they worked independently, and if applicable, details of automation tools used in the process.	5-6
Data collection process	9	Specify the methods used to collect data from reports, including how many reviewers collected data from each report, whether they worked independently, any processes for obtaining or confirming data from study investigators, and if applicable, details of automation tools used in the process.	6
Data items	10a	List and define all outcomes for which data were sought. Specify whether all results that were compatible with each outcome domain in each study were sought (e.g. for all measures, time points, analyses), and if not, the methods used to decide which results to collect.	Table S3
	10b	List and define all other variables for which data were sought (e.g. participant and intervention characteristics, funding sources). Describe any assumptions made about any missing or unclear information.	Table S3
Study risk of bias assessment	11	Specify the methods used to assess risk of bias in the included studies, including details of the tool(s) used, how many reviewers assessed each study and whether they worked independently, and if applicable, details of automation tools used in the process.	6, Table S5
Effect measures	12	Specify for each outcome the effect measure(s) (e.g. risk ratio, mean difference) used in the synthesis or presentation of results.	6

Synthesis methods	13a	Describe the processes used to decide which studies were eligible for each synthesis (e.g. tabulating the study intervention characteristics and comparing against the planned groups for each synthesis (item #5)).	5-6
	13b	Describe any methods required to prepare the data for presentation or synthesis, such as handling of missing summary statistics, or data conversions.	6-7
	13c	Describe any methods used to tabulate or visually display results of individual studies and syntheses.	7-8, Table S4
	13d	Describe any methods used to synthesize results and provide a rationale for the choice(s). If meta-analysis was performed, describe the model(s), method(s) to identify the presence and extent of statistical heterogeneity, and software package(s) used.	6-7
	13e	Describe any methods used to explore possible causes of heterogeneity among study results (e.g. subgroup analysis, meta-regression).	7
	13f	Describe any sensitivity analyses conducted to assess robustness of the synthesized results.	6-7
Reporting bias assessment	14	Describe any methods used to assess risk of bias due to missing results in a synthesis (arising from reporting biases).	Table S5
Certainty assessment	15	Describe any methods used to assess certainty (or confidence) in the body of evidence for an outcome.	Table S6
**RESULTS**			
Study selection	16a	Describe the results of the search and selection process, from the number of records identified in the search to the number of studies included in the review, ideally using a flow diagram.	8, Figure 1
	16b	Cite studies that might appear to meet the inclusion criteria, but which were excluded, and explain why they were excluded.	8
Study characteristics	17	Cite each included study and present its characteristics.	8, Table S4
Risk of bias in studies	18	Present assessments of risk of bias for each included study.	Table S5
Results of individual studies	19	For all outcomes, present, for each study: (a) summary statistics for each group (where appropriate) and (b) an effect estimate and its precision (e.g. confidence/credible interval), ideally using structured tables or plots.	8-10
Results of syntheses	20a	For each synthesis, briefly summarise the characteristics and risk of bias among contributing studies.	Table S6
	20b	Present results of all statistical syntheses conducted. If meta-analysis was done, present for each the summary estimate and its precision (e.g. confidence/credible interval) and measures of statistical heterogeneity. If comparing groups, describe the direction of the effect.	8-10
	20c	Present results of all investigations of possible causes of heterogeneity among study results.	8-10
	20d	Present results of all sensitivity analyses conducted to assess the robustness of the synthesized results.	8-10

Reporting biases	21	Present assessments of risk of bias due to missing results (arising from reporting biases) for each synthesis assessed.	Table S6
Certainty of evidence	22	Present assessments of certainty (or confidence) in the body of evidence for each outcome assessed.	Table S6
**DISCUSSION**			
Discussion	23a	Provide a general interpretation of the results in the context of other evidence.	10-12
	23b	Discuss any limitations of the evidence included in the review.	11-12
	23c	Discuss any limitations of the review processes used.	11-12
	23d	Discuss implications of the results for practice, policy, and future research.	11-12
**OTHER INFORMATION**			
Registration and protocol	24a	Provide registration information for the review, including register name and registration number, or state that the review was not registered.	5
	24b	Indicate where the review protocol can be accessed, or state that a protocol was not prepared.	7
	24c	Describe and explain any amendments to information provided at registration or in the protocol.	5
Support	25	Describe sources of financial or non-financial support for the review, and the role of the funders or sponsors in the review.	7
Competing interests	26	Declare any competing interests of review authors.	12
Availability of data, code and other materials	27	Report which of the following are publicly available and where they can be found: template data collection forms; data extracted from included studies; data used for all analyses; analytic code; any other materials used in the review.	Table S3, 12

From: Page MJ, McKenzie JE, Bossuyt PM, Boutron I, Hoffmann TC, Mulrow CD, et al. The PRISMA 2020 statement: an updated guideline for reporting systematic reviews. BMJ 2021;372:n71. doi: 10.1136/bmj.n71

Table S2. Search strategy for databases

MedLine

No.	Search	Results
1	Extracorporeal Membrane Oxygenation[MeSH] OR Extracorporeal Membrane Oxygenation[Title/Abstract] OR Extracorporeal Membrane Oxygenat*[Title/Abstract] OR Extracorporeal Life Support[Title/Abstract] OR ECMO[Title/Abstract] OR ECLS[Title/Abstract] OR Extracorporeal Circulat*[Title/Abstract] OR Extracorporeal[Title/Abstract]	48,567
2	"SARS-CoV-2"[Mesh] OR "COVID-19"[Mesh] OR "Coronavirus Infections"[MeSH Terms] OR 2019-nCoV[Title/Abstract] OR 2019 ncov[Title/Abstract] OR nCov[Title/Abstract] OR covid-19[Title/Abstract] OR covid19[Title/Abstract] OR COVID-2019[Title/Abstract] OR COVID2019[Title/Abstract] OR sars-cov-2[Title/Abstract] OR "sars cov-2"[Title/Abstract] OR sarscov2[Title/Abstract] OR sarscov-2[Title/Abstract] OR sars-coronavirus-2[Title/Abstract] OR sars corona virus[Title/Abstract] OR sars-like coronavirus[Title/Abstract] OR novel coronavirus[Title/Abstract] OR novel corona virus[Title/Abstract] OR covid*[Title/Abstract] OR coronavirus 2[Title/Abstract] OR coronavirus infection*[Title/Abstract] OR coronavirus disease[Title/Abstract] OR corona virus disease[Title/Abstract] OR new coronavirus[Title/Abstract] OR new corona virus[Title/Abstract] OR new coronaviruses[Title/Abstract] OR novel coronaviruses[Title/Abstract] OR severe acute respiratory syndrome coronavirus-2[Title/Abstract] OR ((coronavirus[Title/Abstract] OR ncov[Title/Abstract] OR sars-cov[Title/Abstract]) AND (2019[Title/Abstract] OR 19[Title/Abstract] OR Wuhan[Title/Abstract]))	230,658
3	#1 AND #2	1,413

Embase

No.	Search	Results
1	‘Extracorporeal oxygenation’/exp OR ‘extracorporeal membrane oxygenation’:ti,ab OR ‘extracorporeal membrane oxygenat*’:ti,ab OR ‘extracorporeal life support’:ti,ab OR ‘ECMO’:ti,ab OR ‘ECLS’:ti,ab OR ’extracorporeal circulat*’:ti,ab OR extracorporeal:ti,ab	78,672
2	'covid 19'/exp OR 'coronavirus disease 2019'/exp OR 'coronavirus infection'/exp OR 'severe acute respiratory syndrome coronavirus 2'/exp OR '2019-ncov':ti,ab OR '2019 ncov':ti,ab OR ncov:ti,ab OR 'covid-19':ti,ab OR 'covid-2019':ti,ab OR 'covid2019':ti,ab OR covid19:ti,ab OR 'sars-cov-2':ti,ab OR 'sars cov-2':ti,ab OR sarscov2:ti,ab OR 'sarscov-2':ti,ab OR 'sars-coronavirus-2':ti,ab OR 'sars corona virus':ti,ab OR 'sars-like coronavirus':ti,ab OR 'novel coronavirus':ti,ab OR 'novel corona virus':ti,ab OR covid*:ti,ab OR 'coronavirus 2':ti,ab OR 'coronavirus infection*':ti,ab OR 'coronavirus disease':ti,ab OR 'corona virus disease':ti,ab OR 'new coronavirus':ti,ab OR 'new corona virus':ti,ab OR 'new coronaviruses':ti,ab OR 'novel coronaviruses':ti,ab OR 'severe acute respiratory syndrome coronavirus-2':ti,ab OR ((coronavirus:ti,ab OR ncov:ti,ab OR 'sars cov':ti,ab) AND (2019:ti,ab OR 19:ti,ab OR wuhan:ti,ab))	251,285
3	#1 AND #2	3,432

Cochrane

No.	Search	Results
1	MeSH descriptor: [Extracorporeal Membrane Oxygenation] explode all trees	193

2	(Extracorporeal Membrane Oxygenation OR Extracorporeal Membrane Oxygenat* OR Extracorporeal Life Support OR ECMO OR ECLS OR Extracorporeal Circulat* OR Extracorporeal):ti,ab	4,763
3	((covid-19 or covid19 or nCov or sars-cov-2 or sarscov2 or sarscov-2 or sars-coronavirus-2 or sars corona virus or sars-like coronavirus or novel coronavirus or novel corona virus or covid* or coronavirus 2 or coronavirus infection* or coronavirus disease or corona virus disease or new coronavirus or new corona virus or new coronaviruses or novel coronaviruses or severe acute respiratory syndrome coronavirus-2) OR ((coronavirus OR ncov OR sars-cov) NEAR/5 (2019 OR 19 OR Wuhan))):ti,ab	9,488
4	(#1 OR #2) AND #3	258

Scopus

No.	Search	Results
1	TITLE-ABS-KEY("Extracorporeal Membrane Oxygenation" OR "Extracorporeal Membrane Oxygenat*" OR "Extracorporeal Life Support" OR "ECMO" OR "ECLS" OR "Extracorporeal Circulat*" OR "Extracorporeal")	81,691
2	TITLE-ABS-KEY(((covid-19 or covid19 or nCov or sars-cov-2 or sarscov2 or sarscov-2 or sars-coronavirus-2 or "sars corona virus" or "sars-like coronavirus" or "novel coronavirus" or "novel corona virus" or covid* or "coronavirus 2" or "coronavirus infection*" or "coronavirus disease" or "corona virus disease" or "new coronavirus" or "new corona virus" or "new coronaviruses" or "novel coronaviruses" or "severe acute respiratory syndrome coronavirus-2") OR ((coronavirus OR ncov OR sars-cov) W/5 (2019 OR 19 OR Wuhan))))	288,721
4	#1 AND #2	2,713

Table S3. Data collection template

Study characteristics: Title, authors, year published, journal, country, hospital, duration of study, inclusion/exclusion criteria

Baseline demographics: Sample size, age, number of male patients, body mass index, comorbidities (hypertension, diabetes mellitus type 2, dyslipidaemia, obesity, chronic obstructive pulmonary disease/asthma, coronary artery disease, smoking, other comorbidities)

Pre-ECMO characteristics: Sequential organ failure assessment score, simplified acute physiology score, acute physiology and chronic health evaluation score, lung injury score, PaO2-to-FiO2 ratio, PaO2, PaCO2, pH, lactate, left ventricular ejection fraction, cardiac arrest, requirement of pre-ECMO renal replacement therapy, D-dimer, fibrinogen, interleukin-6 levels, white blood cell/leukocyte count, time from symptoms to initiation of invasive mechanical ventilation, time from noninvasive ventilation to invasive mechanical ventilation

ECMO characteristics: Indications for ECMO, number of patients receiving venovenous, venoarterial, veno-veno-arterial and veno-arterio-venous, and other types of ECMO, cannulation site, adjuvant therapies including but not limited to: prone positioning, vasodilators, high frequency oscillatory ventilation, paralytics, vasopressors/inotropes, inhaled nitric oxide, steroids, hydroxychloroquine, interleukin antagonists, lopinavir-ritonavir, remdesivir, and other therapies, tine from invasive mechanical ventilation to ECMO cannulation.

Ventilatory characteristics: Tidal volume, fraction of inspired oxygen (FiO2), peak inspiratory pressure, plateau pressure, driving pressure, mean airway pressure, respiratory rate, positive end-expiratory pressure, respiratory system compliance

Outcomes:
Mortality, time to follow-up, survival to discharge, duration of ECMO support, duration of invasive mechanical ventilation, length of hospital and intensive care unit stay, complications while on ECMO (grouped into mechanical, haemorrhagic, neurologic, renal, cardiovascular, pulmonary, infectious, metabolic, limb, thrombotic, and others.

Table S4a. Demographics and outcomes of included studies

Author	Country	Sample size	VV-ECMO	Demographics of ECMO patients			Nonsurvivors	Duration of ECMO (days)	Length of ICU stay	Length of hospital stay (days)
				Male	BMI	Age (yrs)				
Agerstrand 2020	USA	22	15	18		52 (19-68)	10	24.5 (7-74)		
Akhtar 2021	UK	18	16	16	31 ± 6.6	47.3 ± 0.9	4	17.7 ± 9.4		57.2 ± 25.8
Alnababteh 2020	USA	13	13	8	33.39 ± 9.519	58.3 ± 14.54	6	12.85 ± 6.04		
Arachchillage 2021	UK	152	152	114		47 [23-65]	45	17.5 (11-30)		
Barbaro 2021	International (ELSO)	4812	4492	3523	32.28 ± 7.14	49.95 ± 11.97	2411	19.46 ± 12.63*		
Bergman 2021	USA	46	46	38	31.7 ± 6.6	51.3 ± 10	16	22 (13-36)	33 (22-51)	39.5 (29-56)
Bermea 2021	USA	33	33	24	33 (30-35)	53 (42-59)	13	12 (5–23)		
Bohman 2021	USA	22	22	16	31.2 (29.1-37.9)	50.5 (43-56.3)	6	25.75 (12.75-41.25)		
Broman 2021	International (ELSO)	1723					1046			
Cain 2021	USA	18	18	10		51 (44-57)	2		21 (9-36)	25.5 (17-39)
Chandel 2021	USA	24	24	20		46 (37-53)	5	13 (9-21)		
Cheng 2021	China	74		46		63 (55-71)	53	13 (8-21)	18 (10-30.5)	27 (12-39.75)
Correa 2021	Brazil	1296	20				11		36.27 ± 41.9*	45.55 ± 53.24*
Cousin 2020	France	30	30	24	33.33 ± 7.006	55.333 ± 11.676	16	10.667 ± 5.4489		32.22 ± 20.2389
Daviet 2021	France	78	77	59	28.1 (26.1-31.8)	61 (54-64.5)	37	21 (11-31.5)		
Diaz 2021	Chile	85	82	71		48 (41-55)	33		40 (21-57)	50 (24-69)
Dognon 2021	France	50	50	46	31 (28-36)	61 (53-66)	32	12 (7-16)	33 (20-60)	33 (21-64)

Dreier 2021	Germany	16	16	13	27.5 (24.7-32.6)	59 (51-65)	5	36.9 ± 29.7	73.85 ± 52.08	
Durak 2021	Germany	39	38	28	29.7 (26.3-35.2)	56 (50-60)	23	19 (11-29)		
Falcoz 2020	France	17	16	16		56 [30-76]	6	9 [0-16]		
Fang 2021	China	70	70	42			52		28 (15.25-44)	23.5 (16.25-52.25)
Hermann 2022	Austria	101	101	71	31 ± 6	56 ± 10	44	16.4 (8.7-22.7)	31 (20.7-51)	
Huette 2020	France	12	12	12	29.4 (29.2-32.6)	62 (58-64)	4	12 (9-22)		
Jacobs 2021	USA	200	188	138		49.8 ± 12.1	110	19.79 ± 16.08		
Jang 2020	Korea	19	16	15	26.7 (25.6-27.8)	63 (60-66)	10	15.9 (7.7-28.2)		
Jozwiak 2020	France	11	11	7	30 (26-32)	50 (38-59)	6			
Karagiannidis 2021	Germany	3397	3397			57 ± 11	2310			
Kon 2021	USA	27	27	23	32 (29-37)	40 (30.5-47)	1	11 (10-14)		23 (19.5-29)
Lai 2021	China	50	47	34	25.3 (23.1-27.8)	65.3 (58.5-73)	29	17.9 (6-22)		
Lazzeri 2021	Italy	35	35	28	34 ± 6	54 ± 11	18			42 [4-185]
Lebreton 2021	France	302	288	235	29.7 (26.8-33.5)	52 (45-58)	164	14 (8-26)	30 (17-47)	
Li 2021	China	34	31	22		64.5 (56-72)	20			
Loforte 2021	Italy	71	67	61	30.2 ± 6.1	55.4 ± 9.3	45	15 (8-23)		30 (18-45)
Lorusso 2021	International (ELSO)	1531	1383	1209		52.6 [16-80]	672	18		
Murthy 2021	Canada	13	13				9			
Nguyen 2021	USA	1182		844			542		29.1 ± 17.3	36.8 ± 24.9
O'Neil 2021	International (ELSO)	1180	1180	0		49.05 ± 12.66	540			
Olivier 2021	France	56	56	49	30 (27-34)	57 (51-65)	29	18 (10–31)	46 (28-60)	
Rabie 2021	International	307	288	248	28.6 (25.4-33.3)	45 (37-52)	169	15 (9.5-24)		

Raff 2021	USA	32	32	25	35.1 ± 7.8	47.8 ± 10.3	21	12.4 ± 5.7		
Rajajee 2021	USA	23	20	5	33 (27-37)	47 (37-52)	8	16 (8–32)	47 (24-54)	47 (28-54)
Rhoades 2021	USA	31	31	22	32 (21.5-47.6)	53 (32-66)	13			
Riera 2021	International	338	319	258		53 ± 10	156	17 (9-32)		
Roedl 2021	Germany	52	51	34	32.1 (26.3-36.7)	58 (53-64)	35	17 (7–27)	24 (13-44)	
Saeed 2021	USA	292	280	211	32 (29-37)	49 (39-57)	113			
Shah 2021	USA	40	40	33	34 (27-40)	43 (36-50)	19			
Supady 2021	International	127	127	100	29 (26-35)	59 (53-66)	58			
Suwalski 2021	Poland	78	64	60	31.3 ± 9.5	47 ± 11.3	59			
Tabatabai 2021	USA	40	40	32	34 ± 7.1	43.2 ± 8.9	15	29.1± 15.9		55.6 ± 22.5
Takeda 2020	Japan	1190		959			427	15.98 ± 13.78*		
Ulukan 2021	Turkey	22	18	16		56.3 ± 10.6	19	8.5 ± 5.5		
Zhang 2021	UK	56	56	41	29.5 (27-34)	46 (37.5-52.5)	16	13 (8-21)		

Abbreviations: ELSO: Extracorporeal life support organisation, ECMO: extracorporeal membrane oxygenation, ICU: intensive care unit, VV: venovenous, USA: United Statesof America

Data presented as mean ± standard deviation, median (interquartile range), or median [range].
*Values derived from combining subgroup data provided by the studies

Table S4b. Pre-ECMO ventilatory parameters

Author Year	Tidal volume	Peak inspiratory pressure (cm H_2O)	Plateau Pressure (cm H_2O)	Driving pressure (cm H_2O)	Mean airway pressure (cm H_2O)	Respiratory rate	PEEP (cm H_2O)	Compliance (ml/cm H_2O)
Agerstrand 2020	400 (200-600) ml		36 (21-50)			34 (18-38)	15 (10-20)	
Barbaro 2021*		33.9 ± 5.9					13.4 ± 4.0	
Bergman 2021	386 ± 70 ml	34.2 ± 50	30.0 ± 4.4			25.9 ± 6.3	13.7 ± 2.8	
Cheng 2021	5 (5-6) ml/kg		34 (32-35)			30 (28-32)	15 (12-16.5)	
Cousin 2020	6.5 (5.7-6.9) ml/kg		30 (26-31)	15 (13-20)		30 (26-32)	14 (12-16)	
Dognon 2021	260 (180-295) ml		24 (20-26)	12 (10-14)		17 (13-20)	12 (10-14)	21 (14-30)
Daviet 2021	5.8 (5.1-6.2) ml/kg		28 (25-30)			27 (23-30)	12 (9.5-14)	23 (16.7-28.6)
Diaz 2021	5.4 (4.7-6) ml/kg		26.2 (5.2)	15 (14-18)			10.4 (4.1)	22 (18-28)
Dreier 2021	5.7 (4.3–6.3) ml/kg	34.0 (31.0–35.0)				25 (23–25)	15.0 (13.3–15.8)	25.9 (19.4–29.0)
Durak 2021							12 (10-14)	
Falcoz 2020	3.9 ± 1.2 ml/kg		27.75 ± 5.2954	14.75 ± 4.1806		19 ± 4.459	12 ± 2.3	
Fang 2021								
Hermann 2022	6 ± 2 ml/kg	31 ± 5	31 ± 5	18 ± 5		22 ± 5	13 ± 3	
Huette 2020	6.1 ± 0.2 ml/kg		29 ± 1.6		31 ± 1.647	14 ± 1.647		
Kon 2021		31 (28-35)				25 (22-28)	14 (12-16)	
Lai 2021	4.9 (4.4-5.6) ml/kg		30.5 (26.0-34)	13.5 (13-14)		10.3 (10-11)	8.8 (8-10)	
Lazzeri 2021	350 (range 140-420) ml						12 (range 8-16)	
Lebreton 2021	5.6 (4.9-6.2) ml/kg		30 (27-32)	18 (14-21)		28 (26-30)	12 (10-14)	
Li 2021			29.9 ± 9.1				11 ± 2.1	
Loforte 2021	469.6 ± 114.4 ml						13.3 ± 4.1	34.4 ± 18.1
O'Neil 2021*		13.7 ± 3.6		21.36 ± 5.76	22.06 ± 4.57		13.73 ± 3.64	
Ogura 2021							12 (10–15)	
Olivier 2021	5.9 (5.5–6.1) ml/kg		31 (30–34)				10 (6–14)	20 (15–23)
Rabie 2021		36 (34-43)	32 (30-35)	20 (17-23)			13 (10.5-15)	19.5 (15.2-22.8)
Raff 2021								

Rajajee 2021							18 (16–20)	22 (19–26)
Roedl 2021	287 (207–341) ml	30 (25–34)					15 (12–16)	
Shah 2021		38 (34-42)			25 (23-27)			
Supady 2021			32.6 (26.5-35.7)	17.3 (13.3-21.4)		25.5 (21.4-30.6)	14.3 (10.2-16.3)	
Tabatabai 2021		38 ± 7.5			25.1 ± 4.8		15.6 ± 3.5	20.6 ± 7.7
Zhang 2021			30 (27.8-31.2)					

* Values derived by combining subgroup data provided by studies

Table S5. Joanna Briggs Institute Checklist

<u>Case series</u>

Author	Domain										Total score
	1	2	3	4	5	6	7	8	9	10	
Agerstrand 2020	✓	✓	✓	✓	✓	✓	✓	✓	✓	✓	10
Akhtar 2021	✓	✓	✓	✓	✓	✓	✓	✓	✓	✓	10
Arachchillage 2021	✓	✓	✓	✓	✓	✓	✓	✓	✓	✓	10
Bergman 2021	✓	✓	✓	✓	✓	✓	✓	✓	✓	✓	10
Bermea 2021	✓	✗	✗	✗	✗	✓	✓	✓	✓	✓	6
Broman 2021	✓	✓	✓	?	?	✗	✗	✓	NA	✓	5
Cain 2021	✓	✓	✓	?	?	✓	✓	✓	✓	✓	8
Chandel 2021	✓	?	?	✓	✓	✓	✓	✓	✓	✓	8
Daviet 2021	✓	?	?	✓	✓	✓	✓	✓	✓	✓	8
Diaz 2021	✓	✓	✓	?	?	✓	✓	✓	✗	✓	7
Dognon 2021	✓	✓	✓	✓	✓	✓	✓	✓	✓	✓	10
Dreier 2021	✓	?	?	✓	✓	✓	✓	✓	✓	✓	8
Fang 2021	✓	✓	✓	✓	✗	✓	✓	✓	✓	✓	9
Hermann 2022	✓	✓	?	✓	✓	✓	✓	✓	✓	✓	9
Huette 2020	✓	✓	✓	✓	✓	✓	✓	✓	✓	✓	10
Jacobs 2021	✓	✓	✓	✓	✗	✓	✓	✓	✓	✓	9
Jozwiak 2020	✓	✓	✓	✓	✓	✓	✓	✓	✓	✓	10
Karagiannidis 2021	✓	✓	✓	✓	✓	✗	✗	✓	✗	✓	7
Kon 2021	✓	✓	✓	✓	✓	✓	✓	✓	NA	✓	9
Lazzeri 2021	✓	✓	✓	✓	✓	✓	✓	✓	✓	✓	10
Lorusso 2021	✓	✓	✓	?	?	✗	✗	✓	NA	✓	5
Olivier 2021	✓	?	?	✓	✓	✓	✓	✓	✗	✓	7
Rabie 2021	✓	✓	?	✓	✓	✓	✓	✓	✓	✓	9
Rajajee 2021	✓	✓	✓	✓	✓	✓	✓	✓	✗	✓	9
Rhoades 2021	✓	✓	?	✓	✓	✓	✓	✓	✓	✓	9
Riera 2021	✓	✓	?	✓	✓	✓	✗	✓	✓	✓	8
Saeed 2021	✓	✓	✓	✓	✓	✓	✓	✓	✓	✓	10
Shah 2021	✗	✓	?	✗	✗	✓	✓	✓	✗	✓	5
Supady 2021	✓	✓	✓	✓	✓	✓	✓	✓	✓	✓	10
Suwalski 2021	✓	✓	✓	✓	✓	✓	✓	✓	✓	✓	10
Tabatabai 2021	✓	✓	?	✓	?	✓	✓	✓	✗	✓	7
Ulukan 2021	✓	✓	✓	✓	?	✓	✗	✓	✗	✓	7
Zhang 2021	✓	✓	?	✓	✓	✓	✓	✓	✗	✓	8

Domains:

1: Were there clear criteria for inclusion in the case series?

2: Was the condition measured in a standard, reliable way for all participants included in the case series?

3: Were valid methods used for identification of the condition for all participants included in the case series?

4: Did the case series have consecutive inclusion of participants?

5: Did the case series have complete inclusion of participants?

6: Was there clear reporting of the demographics of the participants in the study?

7: Was there clear reporting of clinical information of the participants?

8: Were the outcomes or follow up results of cases clearly reported?

9: Was there clear reporting of the presenting site(s)/clinic(s) demographic information?

10: Was statistical analysis appropriate?

Cohort studies

Author	Domain											Total score
	1	2	3	4	5	6	7	8	9	10	11	
Alnababteh 2020	✓	✓	✓	✓	✗	✓	✓	✗	✓	NA	✓	8
Barbaro 2021	✓	✓	✓	✓	✓	✓	✓	✓	✗	NA	✓	9
Bohman 2021	✓	✓	✓	✓	✗	✓	✓	✓	✓	NA	✓	9
Cheng 2021	✓	✓	✓	✓	✓	✓	✓	✓	✓	NA	✓	10
Correa 2021	✓	✓	✓	✓	✗	✓	✓	✓	✓	NA	✓	9
Cousin 2020	✓	✓	✓	✓	✗	✓	✓	✓	✓	NA	✓	9
Durak 2021	✓	✓	✓	✓	✓	NA	✓	✓	✓	NA	✓	9
Falcoz 2020	✓	✓	✓	✗	✗	✓	✓	✓	✓	NA	✓	8
Jang 2020	✓	✓	✓	✓	✓	✓	✓	✗	✓	NA	✓	9
Lai 2021	✓	✓	✓	✗	✗	NA	✓	✓	✓	NA	✓	7
Lebreton 2021	✓	✓	✓	✓	✓	✓	✓	✓	✓	NA	✓	10
Li 2021	✓	✓	✓	✓	?	NA	✓	✓	✓	NA	✓	8
Loforte 2021	✓	✓	✓	✓	?	NA	✓	✓	✓	NA	✓	8
Murthy 2021	✓	✓	✓	?	?	NA	✓	✓	✓	NA	✓	7
Nguyen 2021	✓	✓	✓	?	?	NA	✓	✓	✓	NA	✓	7
O'Neil 2021	✓	✓	✓	✓	?	NA	✓	✓	✓	NA	✓	8
Raff 2021	✗	✓	✓	✓	✓	NA	✓	✓	✓	NA	✓	8
Roedl 2021	✓	✓	✓	✓	✓	✓	✓	✓	✓	NA	✓	10
Takeda 2020	Not assessable: data extracted directly from Japan ECMONet registry											

Domains:
1: Were the two groups similar and recruited from the same population?
2: Were the exposures measured similarly to assign people to both exposed and unexposed groups?
3: Was the exposure measured in a valid and reliable way?
4: Were confounding factors identified?
5: Were strategies to deal with confounding factors stated?
6: Were the groups/participants free of the outcome at the start of the study (or at the moment of exposure)?
7: Were the outcomes measured in a valid and reliable way?
8: Was the follow up time reported and sufficient to be long enough for outcomes to occur?
9: Was follow up complete, and if not, were the reasons to loss to follow up described and explored?
10: Were strategies to address incomplete follow up utilized?
11: Was appropriate statistical analysis used?

Table S6. Grading of Recommendations, Assessment, Development, and Evaluations

№ of studies	Certainty assessment						Effect			Certainty	Importance
	Study design	Risk of bias	Inconsistency	Indirectness	Imprecision	Other considerations	№ of events	№ of individuals	Rate (95% CI)		
Mortality at longest time of follow up (assessed with: %)											
52	observational studies	not serious	not serious[a]	not serious	not serious	none	-	18211	mean 48.8 % (44.8 to 52.9)	⊕⊕⊕⊕ High	CRITICAL
Mortality for those receiving venovenous extracorporeal membrane oxygenation (assessed with: %)											
44	observational studies	not serious	serious[b]	not serious	not serious	none	-	11992	mean 47.2 % (44.1 to 53.0)	⊕⊕⊕○ Moderate	CRITICAL
Duration of ECMO support (assessed with: days)											
35	observational studies	not serious	serious[b]	not serious	not serious	none	-	9524	mean 16.4 days (14.9 to 17.9)	⊕⊕⊕○ Moderate	IMPORTANT
Duration of intensive care unit stay (assessed with: days)											
14	observational studies	not serious	serious[b]	not serious	not serious	none	-	2095	mean 33.5 days (29.4 to 37.6)	⊕⊕⊕○ Moderate	IMPORTANT
Duration of hospital stay (assessed with: days)											
15	observational studies	not serious	serious[b]	not serious	not serious	none	-	1789	mean 39.2 days (33.0 to 45.4)	⊕⊕⊕○ Moderate	IMPORTANT

Explanations

a. There is significant heterogeneity quantitatively and qualitatively. However, subgroup and regression analysis were able to elucidate covariates responsible for the heterogeneity
b. There is significant quantitative and qualitative variability

Table S7. Summary of subgroup and meta-regression analysis

Subgroup analysis

Subgroup		Studies	Mortality (%)	95% confidence interval (%)
Geographical region<br>$p_{interaction}$ = 0.086	North America	17	41.2	35.2 to 47.5
	Europe	22	50.7	44.6 to 56.9
	Multiple ELSO regions	3	47.9	44.3 to 51.4
	Asia-Pacific	6	58.6	45.3 to 70.7
	Latin America	2	43.9	30.1 to 58.8
	Southwest Asia and Africa	2	71.3	33.5 to 92.5
Timing of last patient enrolment<br>$p_{interaction}$ = 0.0013	1st half 2020<br>(January 1 to May 31)	12	41.2	30.6 to 52.8
	2nd half 2020<br>(June 1 to December 31)	28	46.4	41.9 to 50.9
	1st half 2021<br>(January 1 to May 31)	10	62.0	54.8 to 68.7
	2nd half 2021<br>(June 1 to December 31)	2	46.5	38.8 to 54.4

Meta-regression analysis

Covariate	Studies	B	LCI	UCI	P*
Age	46	0.0443	0.0171	0.0714	**0.0014**
Every 100 days after 1 January 2020	52	0.1611	0.0361	0.2861	**0.012**
Proportion of patients receiving steroids	25	1.2176	0.2494	2.1858	**0.014**
Duration of ECMO	35	-0.0332	0.0663	-0.0001	**0.049**
Time from IMV to ECMO	32	0.0574	-0.0307	0.1454	0.20
BMI	32	-0.0345	-0.1230	0.0540	0.44
Proportion of males	48	-0.2390	-1.2732	0.7952	0.65
SOFA score	23	0.0217	-0.0851	0.1285	0.69
Proportion of patients receiving IL-6A	10	-1.5557	-3.3165	0.2051	0.083
PaO2-to-FiO2 ratio	36	-0.0073	-0.0220	0.0075	0.33
Time from symptoms to initiation of IMV	12	0.0432	-0.0228	0.1092	0.20

Abbreviations: B: regression coefficient, BMI: body mass index, ECMO: extracorporeal membrane oxygenation, IL-6A: interleukin-6 antagonists, IMV: invasive mechanical ventilation LCI: lower confidence interval, P: P-value, SOFA: sequential organ failure assessment UCI: upper confidence interval

*Values in bold indicate p-value < 0.05.

Table S8: Complications reported by included studies

Author	Complication										
	Mechanical	Haemorrhagic	Neurologic	Renal	Cardiovascular	Pulmonary	Infectious	Metabolic	Limb	Others	Patients suffering from ≥1 complication
Agerstrand 2020	12 oxygenator failure	13 bleeding	2 CVA	10 RRT		5 PTX	1 cannula site infection			3 haemolysis	-
Akhtar 2021	6 cannula change<br>5 circuit change		2 ICH								
Alnababteh 2020	2 oxygenator change<br>1 dislodged cannula	7 bleeding		6 RRT		4 pneumonia	2 bacteraemia	1 liver failure<br>2 coagulopathy	2 digital ischemia		
Arachchillage 2021	15 circuit clot	5 BGIT<br>3 major bleed	16 ICH			12 pulmonary haemorrhage				68 venous thrombosis<br>45 PE<br>13 DVT<br>10 combined	-
Barbaro 2021	543 membrane lung failure<br>50 pump failure<br>699 circuit change<br>356 cannula problem<br>174 circuit clot	347 BGIT<br>320 cannula site bleed<br>233 surgical site bleed	31 seizures<br>67 CNS infarct<br>302 CNS haemorrhage<br>52 brain death	453 SCr> 1.5-3 mg/dL<br>217 SCr > 3	443 arrhythmia<br>245 CPR<br>42 tamponade	533 PTX<br>176 pulmonary haemorrhage		301 haemolysis<br>210 hyperbilirubinaemia	15 fasciotomy<br>4 amputation		2090
Bergman 2021				21 RRT							-
Bermea 2021	2 circuit failure	17 bleeding	11 ICH	14 RRT		2 pulmonary haemorrhage				5 VTE<br>2 MOF	-
Bohman 2021	Complications were not reported										NA
Broman 2021	Complications were not reported										NA
Cain 2021	Complications were not reported										NA
Chandel 2021		1 haemoptysis<br>1 BGIT									2
Cheng 2021	2 circuit obstruction<br>1 prolapse	25 BGIT<br>6 surgical site bleed				12 pulmonary haemorrhage	2 SSI<br>13 BSI<br>28 pulmonary infection	7 haemolysis			-
Cousin 2020	6 oxygenator failure	14 cannula site bleed<br>13 major bleed	1 ischemic stroke, 3 haemorrhagic strokes	15 AKI			4 BSI			2 PE<br>3 DVT	

	2 oxygenator thrombosis										
Daviet 2021	11 circuit clot<br>30 circuit change	42 major bleeding		25 RRT			45 VAP<br>33 bacteraemia<br>45 septic shock<br>32CMV<br>21 HSV	34 haemolysis<br>3 HIT		36<br>34 DVT/PE	-
Diaz 2021	20 oxygenator failure	33 bleeding	10 neurologic complications, 11 haemorrhagic stroke			12 PTX	60 HAI<br>44 VAP			19 VTE	-
Dognon 2021	20 circuit change	32 cannula site bleed<br>33 other bleed	2 ischemic stroke<br>6 haemorrhagic stroke	22 RRT	3 CPR	7 PTX	30 BSI<br>33 VAP	11 massive haemolysis		8 thrombosis	-
Dreier 2021	9 oxygenator replacement	1 BGIT	1 ICH			1 PTX				4 acute liver failure<br>1 GI ischaemia<br>6 PE	-
Durak 2021	3 circuit thrombosis	9 mucosal bleed<br>6 cannula site bleed<br>3 BGIT	3 cerebral bleed	28 AKI (stage 2-3)	3 cardiac tamponade	9 endobronchial bleed<br>1 haemothorax				8 PA embolism<br>5 venous thrombosis<br>2 arterial thrombosis	-
Falcoz 2020	2 oxygenator thrombosis	1 haemorrhagic shock<br>61 BGIT<br>5 cannula site bleeding	1 stroke	12 RRT	1 cardiac tamponade		10 VAP			3 thrombophlebitis/PE<br>1 thrombocytopenia	
Fang 2021	Complications were not reported										NA
Huette 2020	3 circuit thrombosis<br>2 RRT circuit thrombosis	3 massive bleeding		11 AKI<br>8 RRT	1 CPR	3 PTX	10 VAP			2 acute liver failure<br>1 HIT<br>1 PE<br>4 DVT	
Jacobs 2021			5 ICH<br>2 CNS injury		6 CPR	1 PTX	7 sepsis			12 MOF<br>8 DIC<br>1 air embolism<br>1 PE	43
Jang 2020			4 hypoxic brain damage<br>1 seizure	9 AKI	1 acute cardiac injury	3 pulmonary haemorrhage<br>3 PTX					
Jozwiak 2020		8 transfusions									
Karagiannidis 2021	Complications were not reported										NA

Kon 2021	1 cannula repositioning	1 bleeding		2 RRT		4 PTX	16 superinfection			5 DVT	11?
Lai 2021	3 thrombi	39 bleeding				3 PTX	16 secondary infection			13 thrombocytopenia	-
Lazzeri 2021				2 AKI needing RRT							2
Lebreton 2021	31 circuit thrombosis<br>39 ECMO/line change	115 major bleed	27 ICH<br>6 ischemic stroke	130 RRT	46 CPR	23 PTX<br>53 PE	257 VAP<br>45 cannula infection			55 Severe thrombocytopenia	-
Li 2021	3 oxygenator failure	4e BGIT	2 ICH<br>1 airway bleed<br>18 other bleed			1 pulmonary haemorrhage<br>4 PTX	8 infectious complications				28
Loforte 2021	10 circuit change	9 major bleed	6 strokes	3 RRT		4 PE	39 pneumonia<br>14 sepsis			2 DVT<br>14 MOF	-
Lorusso 2021	Complications were not reported										NA
Murthy 2021	12 oxygenator failure										12
Nguyen 2021	Complications were not reported										NA
O'Neil 2021	Complications were not reported										NA
Olivier 2021		48 bleed					37 infections			19 haemolysis	29?
Rabie 2021	29 membrane lung failure	73 major bleed<br>36 minor bleed		98 RRT	49 CPR	24 PTX<br>15 PE	214 secondary infections			2 DVT	-
Raff 2021		22 minor bleed<br>3 major bleed								14 MOF	22
Rajajee 2021	Complications were not reported										NA
Rhoades 2021		15 major bleeding		8 RRT						1 portal vein thrombus (age-indeterminate)<br>1 splenic infarct (age-indeterminate)	-
Riera 2021	Complications were not reported										NA
Roedl 2021		50 bleeding							5 non-severe limb ischaemia<br>5 severe limb ischaemia	18 DVT<br>3 PE<br>11 HIT<br>6 DIC	-
Saeed 2021	26 circuit exchange	145 major bleed	17 haemorrhagic stroke<br>4 ischemic stroke	93 RRT			153 secondary infections		7 limb ischemia	42 DVT	-

Shah 2021	Complications were not reported										NA
Supady 2021				73 RRT							73
Suwalski 2021	2 circuit complications	53 massive bleed	5 strokes	24 RRT	16 cardiovascular complications	14 pulmonary complications	33 sepsis	7 metabolic complications	4 limb complications	16 MOF	-
Tabatabai 2021		6 cannula site bleed<br>3 MSK bleed<br>3 intraabdominal bleed<br>7 GU bleed<br>6 BGIT<br>1 cardiac bleed<br>12 auricular/nasopharyngeal bleed	1 ICH				10 pulmonary haemorrhage			17 thrombotic	-
Takeda 2020	Complications were not reported										NA
Ulukan 2021	Complications were not reported										NA
Zhang 2021		1 major bleed	2 ICH<br>2 ischaemic stroke	22 RRT	2 cardiac tamponade	15 PTX				7 MOF<br>15 PE	-

Abbreviations: AKI: Acute kidney injury, BGIT: Bleeding gastrointestinal tract, BSI: Bloodstream infection, CMV: Cytomegalovirus, CNS: Central nervous system, CPR: Cardiopulmonary resuscitation, CVA: Cerebrovascular accident, DIC: Disseminated intravascular coagulation, DVT: Deep vein thrombosis, GI: gastrointestinal, GU: Genitourinary, HIT: Heparin-induced thrombocytopenia, HSV; Herpes simplex virus, ICH: Intracranial haemorrhage, MOF: multiorgan failure, PE: pulmonary embolism, PTX: pneumothorax, RRT: renal replacement therapy, SCr: serum creatinine, SSI: Surgical site infection, VAP: ventialtor-associated pneumonia, VTE: venous thromboembolism.
